# Natural killer cells in cancer immunotherapy

**DOI:** 10.1002/mco2.626

**Published:** 2024-06-15

**Authors:** DanRu Wang, LingYun Dou, LiHao Sui, Yiquan Xue, Sheng Xu

**Affiliations:** ^1^ National Key Lab of Immunity and Inflammation and Institute of Immunology Naval Medical University Shanghai China; ^2^ Shanghai Institute of Stem Cell Research and Clinical Translation Dongfang Hospital Shanghai China

**Keywords:** CAR‐NK, MICA/B, NK cell, NKG2D, tumor immunotherapy, tumor vaccine

## Abstract

Natural killer (NK) cells, as innate lymphocytes, possess cytotoxic capabilities and engage target cells through a repertoire of activating and inhibitory receptors. Particularly, natural killer group 2, member D (NKG2D) receptor on NK cells recognizes stress‐induced ligands—the MHC class I chain‐related molecules A and B (MICA/B) presented on tumor cells and is key to trigger the cytolytic response of NK cells. However, tumors have developed sophisticated strategies to evade NK cell surveillance, which lead to failure of tumor immunotherapy. In this paper, we summarized these immune escaping strategies, including the downregulation of ligands for activating receptors, upregulation of ligands for inhibitory receptors, secretion of immunosuppressive compounds, and the development of apoptosis resistance. Then, we focus on recent advancements in NK cell immune therapies, which include engaging activating NK cell receptors, upregulating NKG2D ligand MICA/B expression, blocking inhibitory NK cell receptors, adoptive NK cell therapy, chimeric antigen receptor (CAR)‐engineered NK cells (CAR‐NK), and NKG2D CAR‐T cells, especially several vaccines targeting MICA/B. This review will inspire the research in NK cell biology in tumor and provide significant hope for improving cancer treatment outcomes by harnessing the potent cytotoxic activity of NK cells.

## Graphical abstract

NK cells have cytotoxic functions against tumor cells especially through NKG2D receptor. This article reviews the mechanisms how tumors escape NK‐mediated killing and therapeutic strategies with NK cells and especially MICA/B–NKG2D axis. Finally, the recent innovative CAR‐NK/NKG2D CAR‐T and MICA/B vaccines in tumor were summarized.

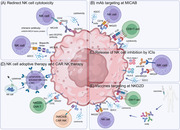

## 1 INTRODUCTION

The innate immunity is the first barrier of protection against pathogens and cancers and is essential for health maintenance. Natural killer (NK) cells, identified in the 1970s,^1^, ^2^ are innate lymphocytes with cytotoxic functions and can impede tumor growth through the process of immune surveillance without the need for prior immune memory and play an essential role in innate immunity.^3^, ^4^ NK cells could recognize target cells with several activating and inhibitory receptors.^5^ Under quiescent conditions, NK cell activity is inhibited by its recognition of MHC class I molecules.^6^ The key feature of NK cell is their cytotoxicity against MHC I‐deficient tumor cells, which has evolved for immune escaping from cytotoxic CD8+ T cells.^7^ These also suggest that NK cells and CD8+ T cells compensate for each other against tumor cells.^8^, ^9^ However, MHC I expressing tumor cells are also killed by NK cells, through their induced expression of certain ligands for activating NK cell receptors, including NKG2D, NKp46, NKp44, and so on.^10^, ^11^, ^12^ Among them, NKG2D ligands MICA/B are upregulated in many types of human tumor cells due to cellular stress such as DNA damage and then activate NK cells for immune surveillance.^13^

The capacity of NK cells to recognize and specifically attack cells under stress or infected by pathogens provides potential therapeutic applications in cancer, infection, and autoimmune diseases.^4^, ^14^, ^15^, ^16^ However, tumors have evolved extremely clever ways to avoid NK cell surveillance, which has resulted in tumor progression and metastases.^17^ Downregulation of ligands for activating NK receptors was the most common strategy employed by tumor cells.^3^, ^18^, ^19^ To avoid these tumors’ immune escape, sophisticated strategies have also been developed to reestablish the effective NK cell surveillance recently, mainly through monoclonal antibodies (mAbs), small molecules, and even vaccination. Cell‐based immunotherapies harnessing the effective NK cell activating receptor NKG2D and NK cell itself have also been developed recently, which have garnered significant interest as an innovative cancer treatment modality. Though there have been reviews on NK cell therapy in tumor,^4^, ^14^, ^20^ these reviews have focused on NK cells, without an emphasis on tumor escaping from NK surveillance and engagement of activating NK receptors.

This review focus on the significant role of the activating NK cell receptor, especially NKG2D and its ligands, on tumor immune escape and NK‐based tumor therapies. We first introduce the historic background of NK cell‐mediated immune responses, further with the characterization of NK cell receptors and ligands, and their signal transduction. Then, we introduce several immune escaping strategies of tumor cells from NK cells and summarize a range of NK cell‐targeted therapeutic approaches such as adoptive NK cell therapy, CAR‐NK, and NKG2D CAR‐T cells, with an emphasis on innovative vaccines targeting MICA/B that bolster the immune response against pan‐cancers. This review would help to improve the knowledge of NK–tumor interaction, promote the realization of NK cell‐based immunotherapy, and provide better therapeutic strategies for tumor patients.

## 2 THE HISTORY AND MILESTONES OF NK CELL IN CANCER IMMUNOTHERAPY

**FIGURE 1:**
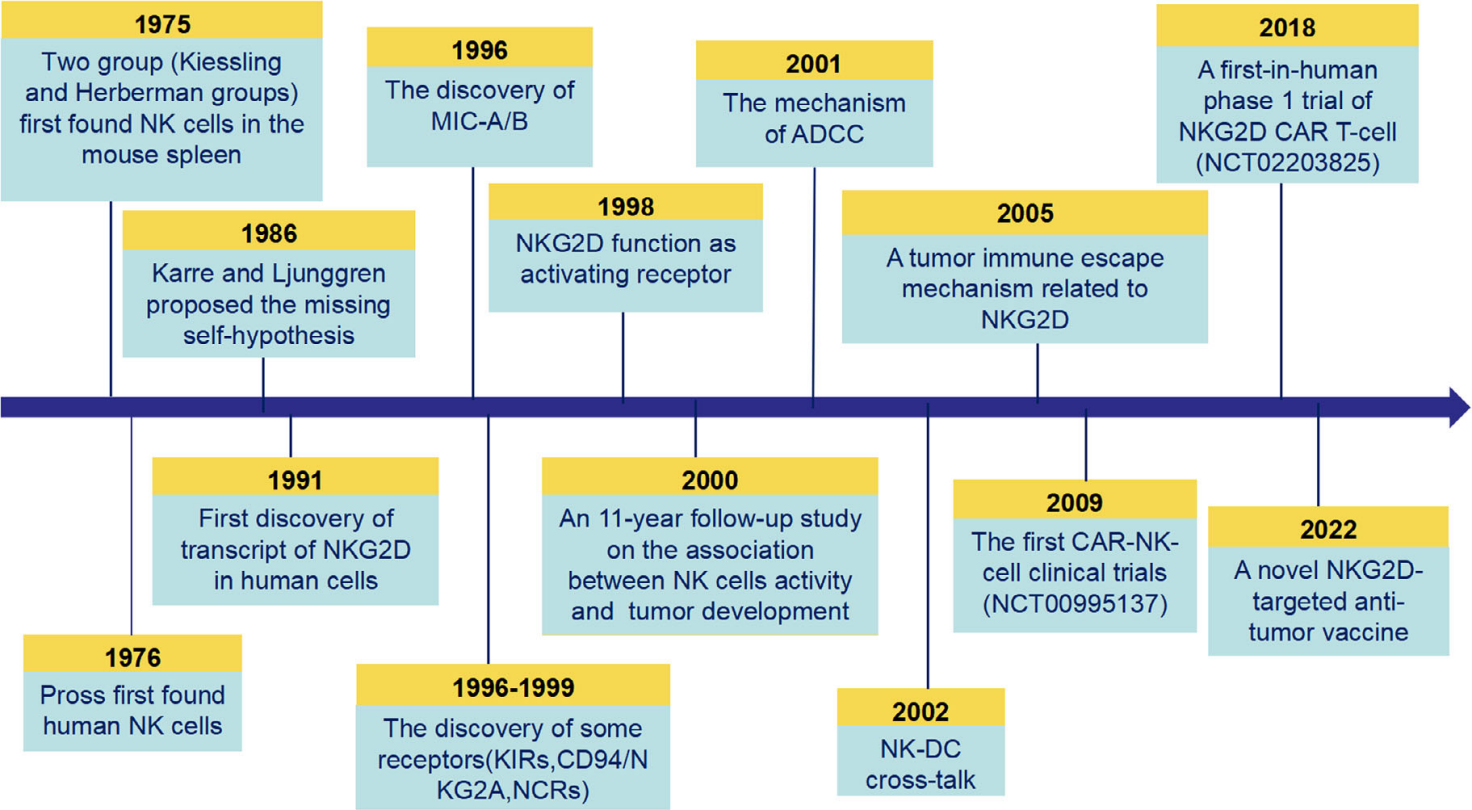
The history and milestones of NK cell in cancer immunotherapy. The timeline illustrates the main discoveries concerning NK cells during a timespan of about 50 years.

NK cells, initially identified in the 1970s subsequent to the discovery of T and B cells in the 1950s (Figure 1), were independently recognized by Kiessling and Herberman in 1975 within the mouse spleen.^1^, ^2^ They characterized these cells as a unique biological entity possessing cytolytic capabilities distinct from other known immune cells, specifically targeting malignant cells. Kiessling et al.^1^ found NK cell‐mediated cytotoxicity against Moloney leukemia virus‐induced leukemia cells. Herberman's group observed high reactivity of them from athymic nude mice against syngeneic and allogeneic tumors cells.^2^ First considered as “background noise” in T‐cell cytolytic assays, NK cells were characterized as cytotoxic effectors of the innate immune system. Then, in 1976, NK cells were discovered in humans as well by Pross and Baines.^21^ According to their ability to lyse tumor cells without prior stimulation, NK cells were initially described as a population of “naturally occurring killer lymphocytes with specificity for tumor cells” on a functional basis.^22^ The majority of these “naturally” cytotoxic cells have a typical and homogeneous morphology and they were hence also referred to as large granular lymphocytes.^23^

A study published in 2000, with a follow‐up duration of 11 years, prospectively monitored the cytotoxic activity of NK cells in a cohort of thousands of individuals, correlating these levels with subsequent tumor development.^24^ The results indicated that innate immune defenses play a critical role in cancer prevention, as evidenced by the association between high cytotoxic activity in peripheral NK cells and a decreased cancer risk, whereas low activity corresponded to an increased risk.^24^ Several subsequent retrospective investigations also proved that NK cell frequency, infiltration in solid tumors,^25^ and function are associated with improved patient survival.^26^, ^27^, ^28^

The discovery of NK cell markers has expanded our understanding of their subsets.^29^ Based on the differential expression of CD56 and CD16 levels, human NK cells are primarily classified into two subsets: CD56^dim^ CD16^+^ NK cells and CD56^bright^ CD16^−^NK cells, which differ in their homing properties.^30^ The majority of circulating NK cells are thought to be the CD56^dim^ subset, which is a mature population. In contrast, the CD56^bright^ subset is less developed, mostly immunomodulatory, and primarily found in secondary lymphoid organs.^31^ What is more, these two subsets have differences in their cytotoxic potential and cytokine production. Highly cytotoxic CD56^dim^ NK cells can express the granzymes (GZMA, GZMB) and perforin (PRF1), and immediately destroy target cells without first priming^32^; CD56^bright^ NK cells are predominately immunoregulatory cells, which has lower cytotoxicity but produce high levels of cytokines including interferon‐γ (IFN‐γ), tumor necrosis factor‐α (TNF‐α), interleukin‐10 (IL‐10), and IL‐13 when exposed to environmental stimuli, such as IL1‐β, IL‐2, IL‐12, IL‐15, and IL‐18.^22^, ^33^

In the late 1980s, Karre et al.^34^ proposed the “missing self” hypothesis, which gave a hint for the molecular characterization of the mechanisms underlying NK‐mediated tumor cell death. This theory explains how NK cells recognize abnormal cells by identifying those who lose or diminish self‐marker. In less than a decade, Moretta's laboratory produced a vast array of mAbs that facilitated the identification and characterization of numerous pivotal receptors, including three non‐HLA class I‐specific activating receptors (NKp46, NKp44, and NKp30) collectively termed as natural cytotoxicity receptors (NCRs),^10^, ^11^, ^35^ the killer immunoglobulin (Ig)‐like receptors (KIRs),^36^, ^37^, ^38^ and CD94/NKG2A.^39^ They also demonstrated how NK cells could destroy target cells by combining signals from inhibitory and activating receptors, through recognizing ligands on tumor or virus‐infected cells, and detecting alterations in HLA class I expression.^40^, ^41^, ^42^ The recognition of stress molecules by NK cells activating receptors leads to the “stress‐triggered self” hypothesis of NK cells.^43^

In 2001, a study found that NK cells quickly activate and degranulate when they identify a target cell covered with a specific antibody, which is called antibody‐dependent cellular cytotoxicity (ADCC).^44^ Thus far, it has been fully appreciated that NK cells can directly kill target cells through the release of cytotoxic granules or by engaging death receptors.^45^ Subsequent investigations revealed that NK cells may not only kill the targeted cells but also incite dendritic cells to polarize and activate the adaptive immune response.^46^, ^47^, ^48^

NK cell receptors play a significant role in cancer immunosurveillance, with the activating NK receptor NKG2D being particularly important in recognizing malignant cells.^49^ The discovery of NKG2D, as a characteristic and novel receptor, dates back to the early 1990s.^50^ In 1998, Bauer et al.^51^ cloned the NKG2D gene for the first time and revealed its important function as an activating receptor. Subsequent research showed that NKG2D specifically recognized its ligands, such as the MHC I polypeptide‐related molecules A (MIC‐A), B (MIC‐B), and MHC‐I‐related molecules UL16 binding proteins (ULBPs).^52^ Furthermore, seminal studies by Spies and colleagues^13^ have revealed the expression of MICA/B on many tumor cell lines and tumor tissues. However, Oppenheim et al.^53^ reported an escape mechanism from NKG2D‐mediated immune responses in 2005, which involves desensitization of the NKG2D pathway via downregulation of NKG2D through repeated stimulation of NKG2D.

Certainly, within the past 10 years, immunotherapy has revolutionized clinical oncology.^54^, ^55^ With the rapid development of chimeric antigen receptor T (CAR‐T) cell therapy, NK cell has gained attention as an alternative to T cell in the field of immune cell engineering because of its intrinsic cytotoxicity, high efficacy and controllable adverse effects.^56^ The first CAR‐NK‐cell clinical trials (NCT00995137) started in 2009, recruited 14 patients under the age of 18 years. CAR‐T cells with chimeric activating receptor NKG2D were also reported. In 2018, the autologous CYAD‐01, a first‐generation NKG2D CAR T‐cell product, was initially tested as a single infusion (NCT02203825).^57^ Following this initial trial, a dose escalation trial, THINK (NCT03018405), demonstrated that CYAD‐01 showed favorable safety data for cancer patients after at least one therapy^58^ (Table 1).

**TABLE 1:** Clinical trials and products associated with NKG2D.

NCT	Drug name	R&D status	Action mechanism	Indication	Drug type
NCT03466320	CYAD‐01	Clinical trial Phase II	NKG2D antagonist	Myelodysplastic syndrome; myeloproliferative diseases; multiple myeloma; acute myeloid leukemia	CAR‐T
NCT03018405			Gene transfer		
			T lymphocyte replacement		
NCT04324996	NKG2D‐ACE2 CAR‐NK cell therapy	Clinical trial Phase II	NKG2D antagonist	Novel coronavirus pneumonia	CAR‐NK
NCT05382377	KD‐025	Clinical trial Phase I/II	NKG2D antagonist	Glioblastoma; colon cancer; hepatocellular carcinoma; medulloblastoma; NKG2DL positive solid tumor	CAR‐T
NCT04550663					
NCT06193902	LEU‐011	Clinical trial Phase I/II	NKG2D antagonist	Solid tumor	CAR‐T
			Gene transfer		
			T lymphocyte replacement		
NCT04167696	CYAD‐02	Clinical trial Phase I	NKG2D antagonist	Myelodysplastic syndrome	CAR‐T
				Recurrent acute myeloid leukemia	
NCT03692429	CYAD‐101	Clinical trial Phase I	NKG2D antagonist	Rectal cancer	CAR‐T
				Unresectable colorectal cancer	
NCT05131763	NKG2D‐based CAR T‐cells	Clinical trial Phase I	NKG2D antagonist	Colon cancer; glioblastoma; liver cancer	CAR‐T
			Immunocytotoxicity		
			T lymphocyte replacement		
NCT04658004	NKG2D CAR‐T‐cell therapy	Early clinical trial Phase I	NKG2D antagonist	Acute myeloid leukemia	CAR‐T
			Gene transfer		
			T lymphocyte replacement		
N/A	LEU‐005	Preclinical	NKG2D antagonist	Solid tumor	CAR‐T
			Gene transfer		
			T lymphocyte replacement		
N/A	LEU‐006	Preclinical	NKG2D antagonist	Hematologic tumor	CAR‐T
			Gene transfer		
			T lymphocyte replacement		
NCT01203631	Tesnatilimab	Clinical trial Phase II	NKG2D antagonist	Alopecia areata; Celiac disease; Crohn's disease; tumor; rheumatoid arthritis	Monoclonal antibody
NCT01181050					
NCT04717999	NKG2D CAR‐T cell therapy	Preclinical	NKG2D antagonist	Glioblastoma	CAR‐T
			Gene transfer		
			T lymphocyte replacement		
NCT05776355	NKG2D CAR‐NK therapy	Clinical trial Phase I	NKG2D antagonist	Acute myeloid leukemia	CAR‐NK
NCT05734898			Gene transfer		
			Natural killer cell replacement		
NCT03370198	CYAD‐203	Clinical trial Phase I	NKG2D antagonist	Colorectal liver metastases	CAR‐T
NCT03310008					
NCT04270461	NKG2D CAR‐NK cells	Clinical trial Phase I	NKG2D antagonist	Metastatic solid tumor	CAR‐NK
NCT03415100			Natural killer cell replacement		
NCT04623944	NKX‐101	Clinical trial Phase I	NKG2D antagonist	Myelodysplastic syndrome; acute myeloid leukemia	CAR‐NK
			IL‐15Rα stimulant		

*Data sources*: clinical registration website.

Recently, NKG2D‐targeted vaccines for cancer immunotherapy have witnessed significant advancements. In 2005, Zhou et al.^59^ demonstrated that coexpression of NKG2D ligands in DNA‐based cancer vaccines effectively enhances their antitumor efficacy by activating both innate and adaptive immune responses. Dana‐Farber Cancer Institute recently has developed a novel antitumor vaccine that activates two main types of immune cells, T cells and NK cells, by targeting MICA/MICB stress molecules.^60^

## 3 NK CELL ACTIVATION AND FUNCTION

NK cells, regarded as innate immune cells, do not go through somatic rearrangement as adaptive immunological T and B cells to produce highly specific receptors that recognize variable antigens.^61^ As the first line of cancer immunosurveillance and early viral immunity, NK cells kill target cells in an antigen‐independent manner.^62^

**FIGURE 2:**
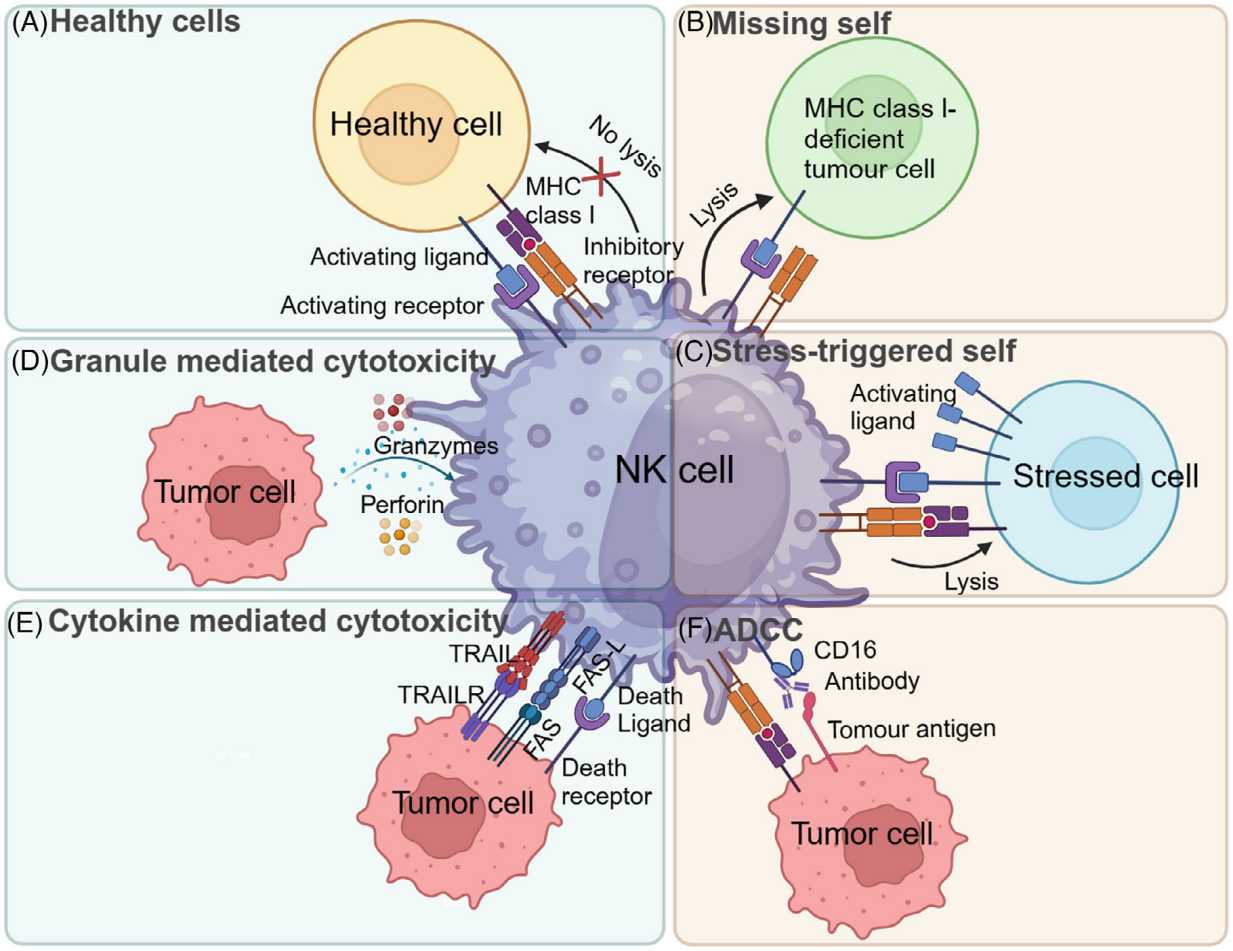
NK cell immune function. (A) NK cell tolerance is a complex integration process of signals from a suite of activating and inhibitory receptors. (B) The missing self‐hypothesis suggested that NK cells sense the absence of MHC and eliminate cells with diminished or absent expression of MHC class I molecules. (C) The stress‐triggered self‐hypothesis of NK cells involves the recognition of disease‐infected or transformed cells (stressed cells) through the upregulation of activating ligands (such as NKG2D). (D) NK cells can release perforin and granzymes to directly killing target cells. (E) NK cells can induce endogenous apoptosis of target cells via the binding of membrane TNF family molecules (FasL, TRAIL, and mTNF) to tumor cell membrane ligands. (F) Antibody‐dependent cell cytotoxicity (ADCC) is exerted by immune cells expressing CD16 receptor against cells coated with antibody, such as virus‐infected or transformed cells.

The mechanisms for NK cells to distinguish healthy cells from target cells form the basis of their functions. NK cell activation is a complex integration process of signals from a suite of activating and inhibitory receptors (Figure 2A), which determines whether an adjacent cell is targeted for killing or not.^63^, ^64^ The “missing self” hypothesis pointed out that NK cells seem to sense the absence of MHC and eliminate cells with diminished or absent expression of MHC class I molecules while MHC I^+^ cells were resistant to lysis^34^ (Figure 2B). This theory marked a significant turning point and sparked more ground‐breaking findings later about the molecular mechanism of NK cell killing. Besides, it has been found that under abnormal condition target cells not only downregulate self‐markers, such as MHC‐class I, but also secrete pathogen‐coded biomolecule^65^ and upregulate self‐produced proteins, which could be recognized by activating receptors on NK cells. Therefore, through their activation receptors, NK cells can kill specific MHC‐I adequate cancer cells by detecting stress‐triggered self‐ligands.^66^ The “stress‐triggered self” hypothesis of NK cells involves the recognition of disease‐infected or transformed cells through the upregulation of ligands for activating receptors, which are not expressed on normal cells^43^ (Figure 2C). A separate mechanism for NK cell activation, termed ADCC, is mediated by the CD16 receptor (also known as FCGR3A), which binds the constant region (Fc) of Igs (Figure 2F). CD16 engagement by Ig‐opsonized cells (cells with antibodies bound to surface membrane antigens) initiates a signaling cascade and kill the antibody‐coated cell.^67^, ^68^

NK cells kill target cells directly through several main mechanisms. First, NK cells can release perforin, which inserts into the plasma membrane and forms pores leading to osmotic lysis, and granzymes, which pass through the pores and activate caspases, inducing apoptosis in target cells^64^ (Figure 2D). Despite the fact that secreted perforin is in close range to both the NK and target cell membranes, the NK cell typically survives due to the protection of densely packed lipid membranes.^69^ Beside the directed release of granules, NK cells can also induce endogenous apoptosis of target cells via the binding of membrane TNF family molecules (FasL, TRIAL, and mTNF) to tumor cell membrane ligands^70^ (Figure 2E). Considering ligation of individual activation receptors (except for CD16) is typically inadequate for provoking cytotoxicity or cytokine release in naive NK cells, it is necessary to preactivate NK cells by exposing them to cytokines such as IL‐2, IL‐12, and type I IFN.^71^, ^72^, ^73^, ^74^

Besides their direct cytotoxic capacity, NK cells can affect the function of other immune cells by secreting a variety of cytokines, chemokines and growth factors, such as IFN‐γ, IL‐13, TNF, FLT3L, C‐C motif chemokine ligand 3 (CCL3), lymphotactin (XCL1), and granulocyte–macrophage colony‐stimulating factor (GM‐CSF).^64^, ^75^ Acting as regulatory cells, NK cells influence various other cell types, such as DCs, T cells, B cells, and macrophages.^32^, ^76^ Upon priming by various soluble factors (for example, IL‐15, type I IFN, IL‐12, IL‐18), NK cells boost the maturation and activation of DCs, macrophages, and T cells, through a combination of cell surface receptors and cytokines.^32^ For example, after activation, NK cells could prime DCs to release IL‐12 and stimulate Th1 responses.^77^ Furthermore, IL‐15 trans‐presentation by IL‐15Rα on DCs could also increases NK cells' capacity to produce IFN‐γ and their cytotoxic activity.^78^ Thus, both DCs and NK cells are regulated by each other through important functional connections.^79^

## 4 ACTIVATING AND INHIBITORY RECEPTORS OF NK CELLS

The activity of NK cells is regulated by an array of cell‐surface receptors that detect the presence of ligands indicative of stress and oncogenic transformation. These receptors can be divided into activating and inhibitory receptors. The balance of activating versus inhibitory signals gives rise to either a tolerance or response to the target cells.^80^

### 4.1 Activating receptors

The activating receptors include the characteristic NCR family (NKp46, NKp30, NKp44), C type lectin family receptor (NKG2D, CD94/NKG2C, CD94/NKG2E, CD94/NKG2F), and KIRs.^5^, ^75^, ^81^ Furthermore, the Fc receptor CD16, which recognizes the Fc part of IgG antibodies, can initiate ADCC.^82^

NCRs, type I transmembrane molecules belonging to the Ig‐like family, include three receptors: NKp30 (also known as NCR3 and CD337), NKp44 (also known as NCR2 and CD336), and NKp46 (also known as NCR1 and CD335).^10^, ^11^, ^35^, ^83^ They are essential for inducing NK cell cytotoxic function against tumors. Their transmembrane domains feature a positively charged amino acid, facilitating their interaction with the transmembrane regions of adaptor proteins TCR‐ζ and/or FcεRI‐γ (for NKp30 and NKp46) or Activating Receptor‐associated Protein (KARAP)/DAP‐12 (for NKp44).^84^ A wide variety of NCR ligands have been reported, including BAT3/BAG6,^85^ MLL5,^86^ and PCNA.^87^

The C‐type lectins are a superfamily of more than 1000 proteins that are defined by having at least one characteristic C‐type lectin‐like domains (CTLDs). They have been subdivided into 17 subgroups on the basis of their phylogeny and domain organization.^88^ Many of them can recognize self (endogenous) and nonself (exogenous) ligands and are involved in a diverse range of physiological functions. Through the binding of MHC class I molecules, C‐type lectins help NK cells recognize cellular transformation and prevent the attack of healthy cells.^89^ The NKG2 receptor family, as an important member of this family, includes seven members referred to as NKG2‐A, ‐B, ‐C, ‐D, ‐E, ‐F, and ‐H, with A/B and E/H being splice variants of the same genes.^50^, ^90^, ^91^ All the molecules encoded by NKG2 gene are expressed on the cell membrane and belong to the type II transmembrane receptor, whose sequence is similar to that of C‐type lectin. These receptors have the effect of inhibiting or activating NK cells. NKG2‐C and potentially ‐E, ‐F, ‐H are the activating family members, which characterized by the presence of a charged amino acid residue in the transmembrane domain mediating interaction with DAP‐12, an adapter molecule containing an immunoreceptor tyrosine‐based activation motif (ITAM).^92^, ^93^ NKG2D, regarded as the best‐characterized activating receptor on NK cells, will be discussed in detail below. In contrast, inhibitory NKG2 proteins (NKG2‐A and ‐B) carry immunoreceptor tyrosine‐based inhibition motifs (ITIMs)^93^, ^94^ and have been found to recognize the same ligand, the nonclassical HLA class I molecule HLA‐E.^39^, ^95^

KIRs have evolved from the Ig‐superfamily and consist of type 1 transmembrane glycoproteins with two or three Ig‐like domains^96^, ^97^ and possess either a short or long cytoplasmic tail. Composed of 14 polymorphic receptors, they are divided into six activating (2DS1−2DS5 and 3DS1), seven inhibitory (2DL1−2DL3, 2DL5 and 3DL1−3DL3), and one (2DL4) that has both activating and inhibitory properties.^98^ KIRs recognize polymorphic HLA‐A, B, and C molecules. The function of activating KIRs in the immune response is partially understood.^99^ Different from inhibitory KIRs, activating KIRs lack ITIM motifs in their cytoplasmic tail and have a transmembrane domain carrying a charged amino acid residue that mediates the association with the ITAM‐bearing molecule KARAP/DAP12.^100^

### 4.2 Inhibitory receptors

Two distinct classes of HLA‐class I‐specific inhibitory receptors are expressed by human NK cells: members of C type lectin family receptor (the CD94/NKG2A) and the inhibitory KIR, mainly including KIR2DL1, KIR2DL2/L3, and KIR3DL1.^5^, ^36^, ^101^, ^102^ Both types of inhibitory receptors contain ITIM motifs in their cytoplasmic tail to transduce inhibitory signals.

Inhibitory KIRs, characterized by 2 or 3 Ig‐like extracellular domains and a long cytoplasmic tail (KIR2DL, KIR3DL), recognize allotypic determinants shared by distinct groups of HLA class I molecules (KIR‐ligands, KIR‐L).^103^ Inhibitory signaling by KIRs is mediated through ITIM, recruiting phosphatases such as SHP‐1. These phosphatases act on proximal kinase signaling pathways involving Vav1 and the adaptor protein Crk.^104^, ^105^, ^106^

The C‐type lectin family member NKG2A is associated with CD94 and can bind to the class‐Ib molecule HLA‐E. It was observed that the expression of NKG2A in NK cells and its ligand HLA‐E in intratumor HCC tissues was increased.^107^ NKG2A‐expressing tumor‐infiltrating nature kill (TINK) cells show signs of fatigued cells and are linked to a poor prognosis.^107^ Unlike KIRs, neither NKG2A nor HLA‐E are polymorphic, which might facilitate the generation of therapeutic agents that block their interaction.^108^

In addition to the HLA‐class I‐specific inhibitory receptors that mentioned above, additional inhibitory checkpoints, such as PD‐1, TIGIT, CD96, TIM‐3, and so on have also been identified in NK cells and are responsible for preserving immune cell homeostasis.^109^ There is growing evidence that NK cells also express PD‐1, PD‐1‐related ICI therapy could also stimulate the antitumor effector actions of NK cell.^110^, ^111^ TIGIT is overexpressed on tired TINK and tumor‐infiltrating T cells in different malignancies in peritumoral lymphocytes, always together with PD‐1 and TIM‐3, and is related to NK cell suppression and functional exhaustion.^112^ In addition to restoring effective tumor immunity and NK cell rejuvenation, TIGIT blockade also improved the effectiveness of ICI treatment against PD‐L1.^113^ CD96 restricts NK cell effector functions via binding to CD155 expressed on tumor cells.^114^ Patients with hepatocellular cancer who exhibit decreased disease‐free survival have malfunctioning (exhausted) TINK cells with elevated CD96 expression; nevertheless, NK cell‐mediated effector capabilities are restored when CD96 is blocked.^115^

### 4.3 Characteristics of NKG2D and its ligands in tumor

#### 4.3.1 Features of NKG2D

The natural killer group 2, member D (NKG2D), a highly conserved C‐type lectin‐like membrane glycoprotein, is a specific cell‐surface receptor, which is only remotely related to the other NKG2 family members and constitutes a separate class of lectin‐like receptors. It can directly bind to a variety of ligand molecular families expressed on the surface of target cells without antigen presentation, thereby activating or costimulating immune effectors,^116^ and then releasing perforin and granzymes to mediate the killing effect.^117^

It is mainly expressed on lymphocytes of the NK cells. It is also found on human naive CD8+T cells, but only express on activated mouse CD8+T cells.^43^, ^118^ In general, CD4+T cells do not express NKG2D even after activation. Expression of NKG2D on NK cells and CD8+ T cells can be modulated by cytokines. In humans, IL‐2, IL‐7, IL‐12, and IL‐15 could upregulate NKG2D expression, whereas TGF‐β, IFN‐β1, and IL‐21 downmodulate NKG2D.^12^ Studies have demonstrated that activating signals mediated by the NKG2D/NKG2DL pathway can override the signals induced by the inhibitory receptors, thereby allowing NKG2D to acts as a “master switch” for activating NK cells.^119^ However, in CD8+ T cells, NKG2D acts as a costimulatory receptor to authenticate the recognition of a stressed target and enhance TCR signaling and T‐cell function.^43^, ^51^, ^120^, ^121^ What is more, NKG2D has potential role in CD8+ T‐cell memory formation, cancer immunity, and autoimmunity.^122^ NKG2D can be expressed on the membrane surface of almost all γδT cells.^123^ It interacts with its ligands and then modulate the cytotoxic capacity of γδT cells.^124^, ^125^, ^126^

The importance of NKG2D in immune surveillance of tumors is highlighted by the observation that NKG2D‐deficient mice are more susceptible to the development of oncogene‐induced tumors,^127^ and tumors expressing endogenous NKG2D ligands or transfected with NKG2D ligands are sensitive to NKG2D‐dependent NK cell‐mediated cytotoxicity in vivo and in vitro.^128^ Besides, the NKG2D pathway can modulate tumorigenesis and tumor progression, which is particularly significant for inhibiting tumor cell metastasis.

#### 4.3.2 NKG2D ligands

There are two main types of NKG2D ligands in human, MICA/B and ULBP1‐6. MICA/B were the proteins encoded by the MHC class I‐chain related genes A and B (MICA and MICB), also called PERB11.1 and PERB11.2, respectively.^109^ Other six ULBPs have a homology with MICA and MICB that is below 25%, also known as retinoic acid early transcripts (RAET).^12^, ^118^ However, there is no MICA/B in mouse cells, and retinoic acid early inducible‐1 (Rae‐1) family of proteins, H60, and murine ULBP‐like transcript 1 (MULT1), which are similar to the MIC protein, serve as NKG2D ligands in mice.^129^

The human MIC genes are located within the MHC class I region of chromosome 6,^130^ among of them, the MICA and MICB are highly polymorphic^131^, ^132^ and are expressed in a codominant manner.^133^ Currently, it is believed that MICA and MICB can be transcribed in 7 members of MIC gene (MICA‐MICG), while the four genes of MICC, MICD, MICE, and MICG are all pseudogenes.^134^ The MICA and MICB proteins encoded by most alleles have similar domain structure to that of classical HLA class I chains, including three extracellular domains (α1−α3), a transmembrane domain and a cytoplasmic domain.^130^ However, unlike their classical HLA class I counterparts, MIC neither binds β2 microglobulin^13^ nor exhibits conventional class I peptide binding.^13^, ^135^

NKG2D ligands are poorly expressed on normal cells but can be induced by cellular stress, including heat shock, viral and bacterial infections, and malignant transformation.^13^, ^136^, ^137^, ^138^ Therefore, they could potentially serve as “danger signals” to alert the immune system the existence of these abnormal cells.^121^, ^138^, ^139^ In healthy individuals, the distribution of MIC was limited to gastrointestinal epithelial cells, endothelial cells and fibroblasts, but the expression levels are low and rare in many cases.^109^ They are upregulated when cells undergo malignant transformation or when they are exposed to other forms of stress such as oxidative stress and viral infection.^32^, ^140^ MICA/B are widely expressed on the surface of tumor cells, including lung, breast, gastric, kidney, ovarian, prostate, colon carcinomas, and melanomas.^51^, ^141^

Increased MICA/B expression in tumor is regulated by the activation of the DNA damage response (DDR) initiated by ATM (ataxia telangiectasia, mutated) or ATR (ATM‐ and Rad3‐related) protein kinases.^142^, ^143^ As the tumor cells have the characteristics of genomic instability and mutagenicity, DDR would be triggered and directly phosphorylate Chk1, Chk2, and so on. The Chk1 would activate transcriptional regulators including the p53 tumor suppressor, p73 and p63, which stimulate the expression of NKG2D ligands.^143^, ^144^ Thus, the pharmacological or genetic inhibition of ATR, ATM, or Chk1 could suppress the downstream mediators of these pathways, and then prevent the upregulation of the NKG2D ligands.^145^ In clinic, some adjuvant treatment options such as chemotherapy, radiation therapy, hormone therapy, and/or immunotherapy can induce or enhance MICA and MICB expression through genomic damage pathways.^146^

MICA/B and sMICA/B, which represent the biological behavior centered on cancer cells and the state of tumor immune surveillance, may have predictive value for cancer patients. Regarding MICA/B expression as identified by immunohistochemistry, a higher level of MICA/B expression was linked to a longer survival in gastrointestinal malignancies. But when all cancer types were taken into account, there was no statistically significant difference seen for the MICA/B expression level.^147^ In addition to the membrane‐bound form, a soluble isoform of MICA/B (sMICA/B) exists in the serum. In comparison with MICA/B, soluble MICA/B is a more accurate prognostic predictor.^147^ There is a negative correlation between sMICA/B levels and patient prognosis, and higher levels predict poorer outcomes.

The type I membrane glycoprotein UL16, which is only expressed in HCMV‐infected cells and not in viral particles, is encoded by the human cytomegalovirus (HCMV).^148^ Cosman et al.^129^ discovered and named two ULBPs, ULBP1 and ULBP2, using UL16‐FC fusion proteins. ULBP is a class of human cell surface molecules. There have been four ULBP molecules found thus far, which are ULBP1, ULBP2, ULBP3, and ULBP4. According to assessments of amino acid sequences, ULBPs and MICA are 23−26% similar. They also feature α1 and α2 domains, just like MHC‐I molecules, but they lack α3 domains,^129^ do not bind β2 microglobulin, and do not have peptides.^132^ ULBP1, ULBP2, and ULBP3 are GPI‐linked membrane proteins, while ULBP4 is a transmembrane protein.^149^, ^150^, ^151^ Murine ULBP‐like transcript 1 (MULT1) was also discovered in 2002. Although it has a large intracellular domain, its sequence bears a strong resemblance to ULBP3.^152^

In contrast to MIC, ULBP is expressed more broadly in a range of normal tissues as well as malignancies. Numerous normal organs, including the heart, lung, testis, bone marrow, and thymus, have been demonstrated to express ULBP mRNAs.^129^ Crucially, HCMV‐infected cells can generate ULBP1, ULBP2, and ULBP3.^153^, ^154^ Consequently, ULBP is crucial to the process of viral infection and the escape of the HCMV from immune surveillance.^153^

David Cosman and Marek Kubin found that tumor cells that were resistant to NK cells could be effectively lysed when transfected with ULBPs, and that the pathway was dependent on NKG2D.^129^, ^149^ All of this suggests that ULBPs may have roles in the immune system's defense against viruses and cancer. NK cell production of cytokines and chemokines is stimulated by ULBPs, and NK cell cytotoxicity is conferred onto NK cell‐resistant target cells that express ULBPs.^155^ What is more, according to earlier studies, free soluble ULBPs (sULBPs) can activate NKG2D, causing NK cells to release cytokines such IFN‐γ, TNF‐α, and MIP‐1β.^156^

#### 4.3.3 Signal transduction through NKG2D

**FIGURE 3:**
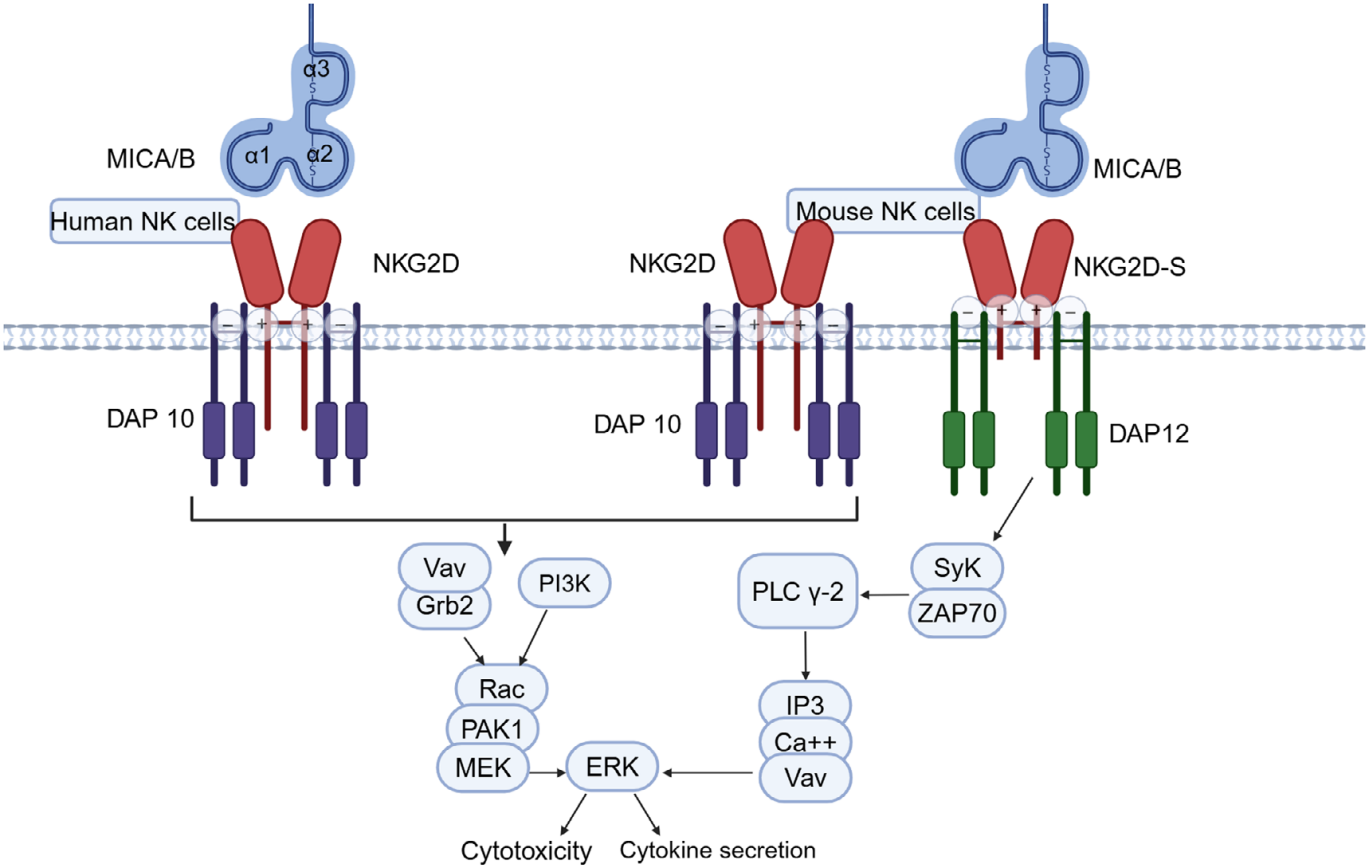
MICA/B‐NKG2D signaling in NK cells. MICA/B on tumor cells can be recognized by NKG2D. NKG2D functions by binding to DAP10 and DAP12 through a salt bridge formed by opposing charged residues in their transmembrane domain. The cytoplasmic domain of DAP10 has a YxxM motif, which recruits the p85 PI3K subunit and Grb2. Besides, an NKG2D isoform generated by alternative splicing can also associate with DAP12 in activated mouse NK cells. DAP12 has an ITAM in its cytoplasmic domain, which recruits and activates the Syk and ZAP70 protein tyrosine kinases. These two signaling pathways ultimately lead to cytokine production and cytotoxicity of NK cells.

NKG2D is a homologous dimer composed of two disulfide bonded transmembrane proteins with a very short intracellular domain and no signal transduction properties.^50^ In mouse and human cells, stable surface expression of NKG2D requires a complex formation of NKG2D dimer with a Tyr‐X‐X‐Met (YXXM) adaptor signaling molecule DAP10.^128^ The NKG2D–DAP10 receptor complex is expressed on the cell surface as a hexamer, with 2 NKG2D and 4 DAP10 molecules (Figure 3). Each subunit of NKG2D noncovalently associated with two DAP10 disulfide‐bonded homodimers. This association occurs by interactions between their transmembrane domains through a salt bridge formed by opposing charged residues.^157^, ^158^ Upon ligand engagement of NKG2D, DAP 10 is phosphorylated by src‐family kinases, which permits the recruitment of the p85 phoshoinositide‐3 kinase (PI3) subunit and the signaling intermediate Grb2‐Vav 1 to fully activate NK cell cytotoxic pathways.^159^

Specifically, activated mouse NK cells also express a splice variant NKG2D‐S, which is 13 aa shorter than normal NKG2D and signals through either DAP10 or the ITAM‐containing adaptor molecule DAP12,^160^, ^161^ which, after phosphorylation, recruits and activates ZAP70 and Syk.^162^Therefore, in activated mouse NK cells, NKG2D‐dependent activation uses both the PI3K and the Syk/ZAP70 pathway through DAP10 and DAP12, respectively, while in human NK cells only the PI3K kinase pathway through DAP10 is engaged (Figure 3).

## 5 MECHANISMS OF TUMOR IMMUNE ESCAPE FROM NK CELL

### 5.1 Downregulation of ligands for activating NK cell receptors

NK cells express a variety of activating receptors, such as NKG2D, NKp44, NKp46, and NKp30,^5^, ^75^, ^81^ which initiate NK cell killing when engaged by their ligands on tumor cells. These ligands are typically stress‐induced proteins or molecules that are upregulated on infected or transformed cells. Tumor cells can downregulate or lose expression of these ligands and prevent the engagement of activating receptors on NK cells, leading to reduced NK cell activation and impaired tumor cell killing.

As the most studied activating receptor of NK cells, NKG2D plays a key role in tumor escaping from NK cells. Thus, we take the NKG2D for example,^51^, ^120^, ^121^ and introduce various mechanisms that tumor cells could escape from NKG2D recognition.

#### 5.1.1 Proteolytic shedding of MICA/B

**FIGURE 4:**
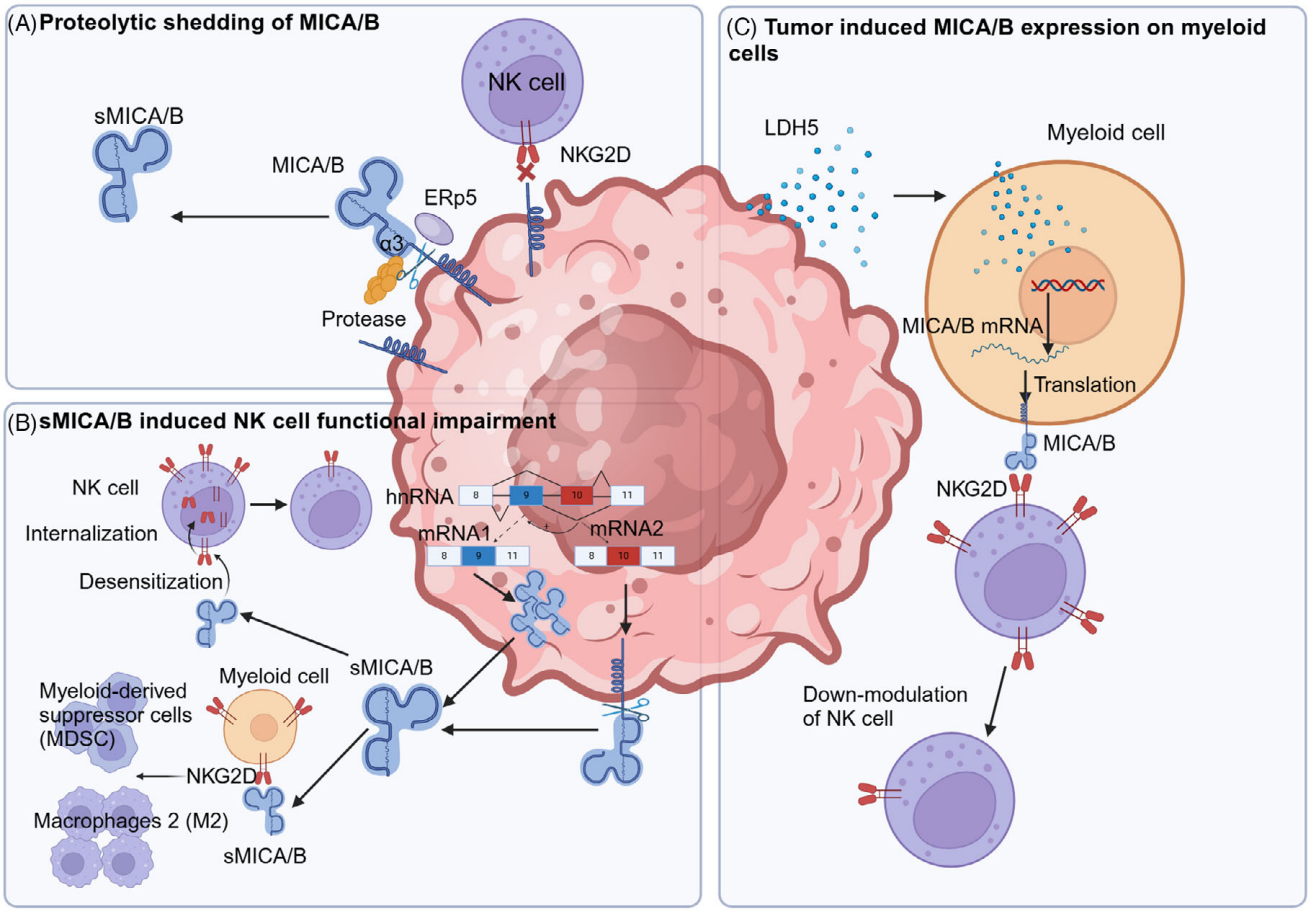
Mechanisms of tumor immune escape associated with MICA/B. (A) Proteolytic shedding of MICA/B. Shedding of surface MICA/B by protease makes NK cells unable to recognize tumor cells. MICA/B shedding is initiated by binding of disulfide isomerase ERp5. Upon the unfolding by ERp5, metalloproteases (MMP14, ADAM10, ADAM17, etc.) cut MICA/B somewhere in the stalk, thus releasing the entire extracellular portion of MICA/B. (B) sMICA/B induced NK cell functional impairment. Large amounts of sMICA/B in the tumor microenvironment desensitizes NK cells and promotes suppressive myeloid cells. sMICA/B could be generated through alternative splicing or shedding of membrane MICA/B. These sMICA/ B binds to NKG2D on the surface of NK cells, desensitizing NK cells by NKG2D internalization. It can further promote the expansion of MDSC and skew M2 macrophages generation by directly act on NKG2D expressed on these myeloid cells. (C) Tumor induced MICA/B expression on myeloid cells. The release of LDH5 by tumor cells induce the expression of MICA/B on the surface of myeloid cells, which causes the downmodulation of NKG2D on NK cells, preventing their recognition of NKG2D ligand‐bearing tumors.

The ectodomains of MICA/B consist of three C‐type Ig‐like domains termed α‐1, α‐2, and α‐3 domains.^163^ The α‐1 and α‐2 domains are relatively distant from the cellular membrane and serve as NKG2D binding sites, whereas the membrane‐proximal α‐3 domain is responsible for the proteolytic cleavage.^163^ The linear stalk in‐between the α‐3 domain and the transmembrane domain have putative proteolytic cleavage sites.^164^ MICA/B shedding is a multistep process that initiated by ERp5 and subsequently sliced by metalloproteases (MPs) (Figure 4A). The disulfide isomerase ERp5 removes the disulfide bond between the amino acid residues 202 and 259 in α‐3 domain.^165^ The removal of this disulfide bond likely unfolds the α‐3 domain and exposes the proteolytic cleavage site. Then, MPs, including MMP14, ADAM10, and ADAM17,^166^, ^167^ cut MICA/B somewhere in the stalk close to the α‐3 domain, and release the entire extracellular portion of MICA/B^3^.It is suggested that these MPs were activated by cytokine pathways. TGF‐β1 negatively affects the expression of these NKG2DL on tumor cell surface by promoting the expression of MMPs, which leads to the MICA abscission. The shedding of MICA/B could also form soluble MICA/B, which function to desensitization of NK cells (detailed in Section 5.3).

#### 5.1.2 Inhibiting MICA/B transcription and translation

Numerous ways by which tumor cells suppress the transcriptional expression of the MICB ligand have been discovered. microRNAs (miRNAs) are able to posttranscriptionally control the expression of ligands. Furthermore, genetic changes such as epigenetic modifications can also block the expression of the ligand.

MiR‐10b, a metastasis‐associated miRNA, has been found to directly bind to the 3′ untranslated (UTR) region of MICB, leading to its downregulation, then diminish NKG2D recognition.^168^ Additionally, nine novel miRNAs (miR‐320c, miR‐320a, miR‐320b, miR‐320c, miR‐320d, miR‐542‐3p, miR‐641, miR‐661, and miR‐940) have been identified as posttranscriptional regulators of MICB expression through both the 3′‐UTR and 5′‐UTR.^169^ This process may involve epigenetic modifications, as suggested by the upregulation of miR‐127, a potential tumor suppressor, by chromatin‐modifying drugs.^170^ Furthermore, the role of aberrant DNA hypermethylation in the regulation of miRNA expression in cancer has been explored, indicating a potential mechanism for the downregulation of MICB.^171^

MiR‐10b, a metastasis‐associated miRNA, has been found to directly bind to the 3′‐UTR region of MICB, leading to its downregulation, then diminish NKG2D recognition.^168^ Additionally, nine novel miRNAs (miR‐320c, miR‐320a, miR‐320b, miR‐320c, miR‐320d, miR‐542‐3p, miR‐641, miR‐661, and miR‐940) have been identified as posttranscriptional regulators of MICB expression through both the 3′‐UTR and 5′‐UTR.^169^ This process may involve epigenetic modifications, as suggested by the upregulation of miR‐127, a potential tumor suppressor, by chromatin‐modifying drugs.^170^ Furthermore, the role of aberrant DNA hypermethylation in the regulation of miRNA expression in cancer has been explored, indicating a potential mechanism for the downregulation of MICB.^171^

### 5.2 Overexpression of ligands for inhibiting NK cell receptors

In addition to avoid from activating NK receptors, tumor cells can upregulate the expression of inhibitory ligands, such as HLA‐G (ligand for KIR), HLA‐E (ligand for NKG2A/CD94), or PD‐L1, which engage inhibitory receptors on NK cells, resulting in the inhibition of NK cell cytotoxicity.^172^

HLA‐G, a nonclassical human leukocyte antigen, may be a predictive marker for certain malignancies due to its association with suppressed immune response and malignant transformation.^173^ Immune evasion and tumor progression is further facilitated by HLA‐G overexpression in various tumors and its interaction with KIRs on immune cells.^174^, ^175^ This overexpression is influenced by epigenetic mechanisms (DNA methylation and histone modifications),^176^, ^177^ and tumor microenvironmental factors,^178^, ^179^, ^180^ particularly hypoxia, which stabilizes the hypoxia‐inducible factor 1 (HIF‐1α) and other factors, leading to increased HLA‐G expression.^181^ Accordingly, a particular hypoxia responsive element (HRE) in exon 2 are required for HLA‐G overexpression in glioma cells.^182^ Stabilized HIF‐1α translocates into the nucleus under hypoxic circumstances, where it binds to HIF‐1β. HIF1α/β then activates transcription through recognizing HREs.^183^ Furthermore, the response may be amplified by a polymorphism HRE at −966 bp in the 5′UT region.^182^ These results demonstrate the potential of HLA‐G as a therapeutic target for cancer.

Numerous variables influence the upregulation of HLA‐E. HCMV has been shown to increase HLA‐E surface expression, possibly as a means of immune evasion.^184^ Marín et al.^172^ further showed that the availability of free β2‐microglobulin in tumor cells, especially those with HLA‐class Ia downregulation, is correlated with HLA‐E expression. This implies that HLA‐E may play a part in tumor immune escape.^172^ Hofer et al.^185^ demonstrated that hypoxia can increase the expression of erythroid 5‐aminolevulinate synthase, a heme biosynthesis‐related enzyme that may have an indirect effect on the expression of HLA‐E.^185^ All of these findings point to the complexity of HLA‐E upregulation as a process that is impacted by cellular stress, tumor cell properties, and viral infection.

The upregulation of PD‐L1 in tumor cells is a complex process involving various mechanisms. Concha‐Benavente et al.^186^ highlight the role of JAK/STAT signaling pathways, IFN‐γ, and specific receptors like the epidermal growth factor receptor (EGFR) in inducing the expression of PD‐L1. NF‐κB, a master transcription factor of inflammation and immunity, is emerging as a key positive regulator of PD‐L1 expression in cancer.^187^ NF‐κB directly induces PD‐L1 gene transcription by binding to its promoter, and it can also regulate PD‐L1 posttranscriptionally through indirect pathways. Scientists also discovered that truncating the PD‐L1 3′UTR can alleviate the PD‐L1 suppression caused by miRNA, resulting in its overexpression.^188^ What is more, epigenetic modifications, such as DNA methylation and histone modifications are also involved in regulating PD‐L1 expression.^189^

### 5.3 Production of immunosuppressive factors

Immunosuppressive tumor microenvironment is created by the secretion of immunosuppressive substances by tumor cells, such as TGF‐β, IL‐10, indoleamine 2,3‐dioxygenase (IDO), PGE2, or adenosine. These elements may directly hinder the function of NK cells or recruit immune‐suppressive cells that block NK cell activity, such as regulatory T cells (Tregs) or myeloid‐derived suppressor cells (MDSCs).^190^, ^191^

TGF‐β1 is a secretory immune‐suppressive characteristic shared by Treg and TAM cells in the tumor microenvironment,^192^ which causes the downregulation of NKG2D on NK cells and CD8+ T cells in the tumor microenvironment.^193^, ^194^ When NK cells are activated, TGF‐β1 limits the production of IFN‐γ, suppresses cytotoxic activity, hinders the release of cytotoxic granules, and lowers the expression of activating receptors that are cytotoxic.^195^, ^196^, ^197^, ^198^ IL‐10 encourages the development of Tregs, and has similar immunosuppressive effects on NK cells.^190^, ^191^

PGE2 and L‐kynurenine (the tryptophan catabolite generated from the IDO‐1) also have immunomodulatory properties. The expression and function of various activating NK receptors, including NKp46, NKp44, and NKG2D, are significantly impacted by both factors.^199^, ^200^ Specifically, DC that express IDO have a profoundly suppressive effect on the immune system by influencing the growth and effector capabilities of NK cells as well as triggering the transformation of CD4+ T cells into Treg.^201^ Both adenosine (an endogenous purine nucleoside that is highly produced by tumors expressing CD39 and CD73) and macrophage migration inhibitory factor (also known as glycosylation‐inhibiting factor)^202^ have been shown to inhibit cytotoxicity and cytokine production in human NK cells. The former is primarily due to the engagement of the adenosine receptor 2A (AdoR2A) on NK cells, which is coupled to adenylyl cyclase via Gs protein.^203^, ^204^

Soluble NKG2D ligands have been detected in the serum of patients with multiple types of cancer.^205^ Soluble MICA/B could be generated through shedding of membrane MICA/B by certain proteases. Furthermore, the soluble NKG2D ligands could also be generated by alternative splicing of certain MIC genes, resulting in the generation of transcripts lacking a transmembrane and cytoplasmic domain that finally produce a soluble MICA/B protein.^128^ These soluble NKG2D ligands (sMICA and sMICB) can bind to NKG2D, lead to downmodulation of NKG2D and subsequent functional impairment of NKG2D‐dependent activation, and finally facilitate tumor progression^206^, ^207^, ^208^, ^209^, ^210^, ^211^ (Figure 4B). Other studies also reveal that sMICA/B can also facilitate MDSCs differentiation and expansion through directly activates NKG2D on myeloid cells.^212^ In addition, sMICA/B further skews macrophages to the suppressive alternative phenotype through activation of STAT3.^212^ Thus, through suppression antitumor immunity and exacerbating tumor suppressing cells, sMICA/B promotes tumor progression. Clinical data demonstrated that higher serum soluble MICA of melanoma patients indicate less benefits following immunotherapy with T‐cell checkpoint blockade, indicating that MICA/B shedding is a new therapeutic target in cancer immunology.^213^

Beside tumor cells, MICA/B could be expressed on immune cells in the TME. Lactose dehydrogenase (LDH) 5 released from tumor cells can induce expression of NKG2D ligands on the surface of monocytes.^127^ Expression of NKG2D ligands by myeloid cells causes the downmodulation of NKG2D on NK cells, preventing their recognition of NKG2D ligand‐bearing tumors, and finally impairs their ability to attack and eliminate tumors, facilitates tumor immune escape (Figure 4C). In clinic, monocytes are found to express NKG2D ligands in patients with several types of cancer including glioblastoma, breast cancer, prostate cancer, and hepatocellular carcinomas,^127^ suggesting it may represent a common mechanism of immune evasion. Blocking LDH5 to preserve the lethality of NK cells may improve the survival of cancer patients.

Due to the suppressive milieu these immunosuppressive substances produce, NK cells' ability to perform effector functions is compromised, which allows tumor cells to evade detection and destruction. In order to restore NK cell function and boost antitumor immune responses, strategies for countering the effects of these immunosuppressive substances are being investigated.

### 5.4 Resistance to NK cell‐mediated killing

Tumor cells can even avoid NK cell effector activity following target cell recognition.^214^ All forms of cancer have different mechanisms that impart apoptotic resistance, impacting both the intrinsic (via mitochondria) and extrinsic (through death receptors) pathways.^215^ The primary mechanisms of resistance to NK cell‐related apoptosis include genetic background and modified expression patterns of pro‐ and antiapoptotic proteins.

Throughout the course of cancer development, tumor cells experience a wide range of genetic and epigenetic changes that impact the genes regulating apoptotic signaling pathways at various levels.^216^ One typical method of inhibiting tumor cell death is to disrupt caspase activity, usually by genetic alterations.^214^ For example, human malignancies have significant levels of caspase‐8 mutations.^217^ Molecules that inhibit the apoptotic cascade may exhibit aberrant activity in tumor cells, rendering them resistant to planned cell death. Furthermore, resistance to NK cell‐mediated apoptosis is caused by tumor cells' downregulation or inhibition of proapoptotic proteins. Colon and stomach tumors with microsatellite instability have been shown to harbor frameshift mutations that inactivate the proapoptotic protein Bax.^218^

The engagement of death receptors, which transduce the death signal to intracellular components of the route, is necessary for the activation of the extrinsic apoptotic cascade. As a result, when these receptors are inactivated, apoptosis is dysregulated, which is a tactic connected to the development of tumors. In a range of solid tumors and hematological malignancies, high expression of antiapoptotic proteins such as c‐FLIP, which impedes TRAIL‐mediated apoptosis, has been linked to apoptosis resistance and a poor prognosis.^219^, ^220^, ^221^ In a PRF1‐deficient mouse model, c‐FLIP overexpression prevented tumor cells from being lysed by NK cells in vivo, emphasizing the significance of this protein's function in malignancy.^222^

Furthermore, tumor cells have developed unique tactics to obstruct the activity of granzymes and/or perforin, which allows for immune escape.^214^ Such NK cell sculpting can be accomplished directly by means of soluble substances released by cancer cells, for example, or indirectly by recruiting suppressor cells that obstruct NK cell antitumor action. MDSCs from mice having mammary cancer were shown to lower PRF1 levels in NK cells through coculture tests, which was correlated with a decrease in NK cell cytotoxicity in vivo.^223^ Besides, the unfavorable tumor microenvironment could obstruct the degranulation process, actively promoting tumor resistant to NK cell‐mediated apoptosis. The activation of autophagy in hypoxic human breast cancer cells was found to enhance GZMB breakdown. The killing ability of NK cells in vivo was restored by inhibiting autophagy by specifically targeting beclin1, a crucial regulator of autophagosome formation. Additionally, the presence of GZMB in hypoxic tumor cells in vitro was also restored.^224^, ^225^ Various GZM family members are suppressed by serine protease inhibitors. Protease inhibitor 9 (Serpin B9) primarily targets GZMB's proteolytic action. Tumor cell lines expressing Serpin B9 demonstrated greater resistance to GZMB‐induced apoptosis, suggesting that this evasion tactic may reduce NK cells' capacity to kill tumors by blocking the degranulation pathway.^226^ The effectiveness of ADCC‐based therapeutics is restricted by resistance mechanisms that target PRF1 and GZM, as ADCC‐mediated cytotoxicity is dependent on these immune mediators.^227^, ^228^

Chronic stress conditions like hypoxia or oxidative stress are often seen in the TME, and they can have a deleterious effect on the antitumor function of NK cells directly or through other cell subsets.^229^ Apoptosis is regulated by a few key proteins, and hypoxia tips the scales in favor of an antiapoptotic cellular state. Reduced amounts of proapoptotic BCL2 family members, like Bax, are seen in hypoxic tumor cells.^230^ In contrast, hypoxic tumor cells have higher levels of a number of antiapoptotic proteins, such as Mcl‐1 and c‐IAP2.^231^, ^232^ It's interesting to note that cancer resistance to NK cell‐mediated apoptosis is influenced by nonimmune cells from the tumor location, such as CAFs.^233^

## 6 NK CELL‐BASED IMMUNOTHERAPEUTIC STRATEGIES

Nowadays, only a subset of patients responds to current immunotherapy mediated by immune checkpoint inhibition, and many responders acquire resistance after initial responses.^234^, ^235^, ^236^ Immune checkpoint inhibition relies on the tumor expression of peptide–MHC complex on tumor cells. Those tumors possess impaired antigen presentation would fully escape the killing by CD8+ T cells. Thus, alternative immunotherapies with distinct mechanisms could effectively treat the resistant patient population.

Nearly 20 years ago, NK cell‐based immunotherapy of cancer emerged as an effective and safe treatment approach for patients diagnosed of leukemia.^237^ Despite NK cell therapies' lower level of clinical success when compared with T cell therapy, their early preclinical and clinical successes have sparked growing interest in their potential. Various strategies include redirecting NK cell activity against tumor cells, releasing inhibitory signals that limit NK cell function, creating large‐scale NK cells for adoptive transfer and cultivating an environment beneficial to NK cell activity.

### 6.1 Engagement of activating receptors on NK cells

To increase NK cell activity in vivo, a variety of activating and costimulatory receptors expressed by NK cells like CD16 and NKG2D can be targeted with antibodies, soluble ligands, and other bioactive compounds.

**FIGURE 5:**
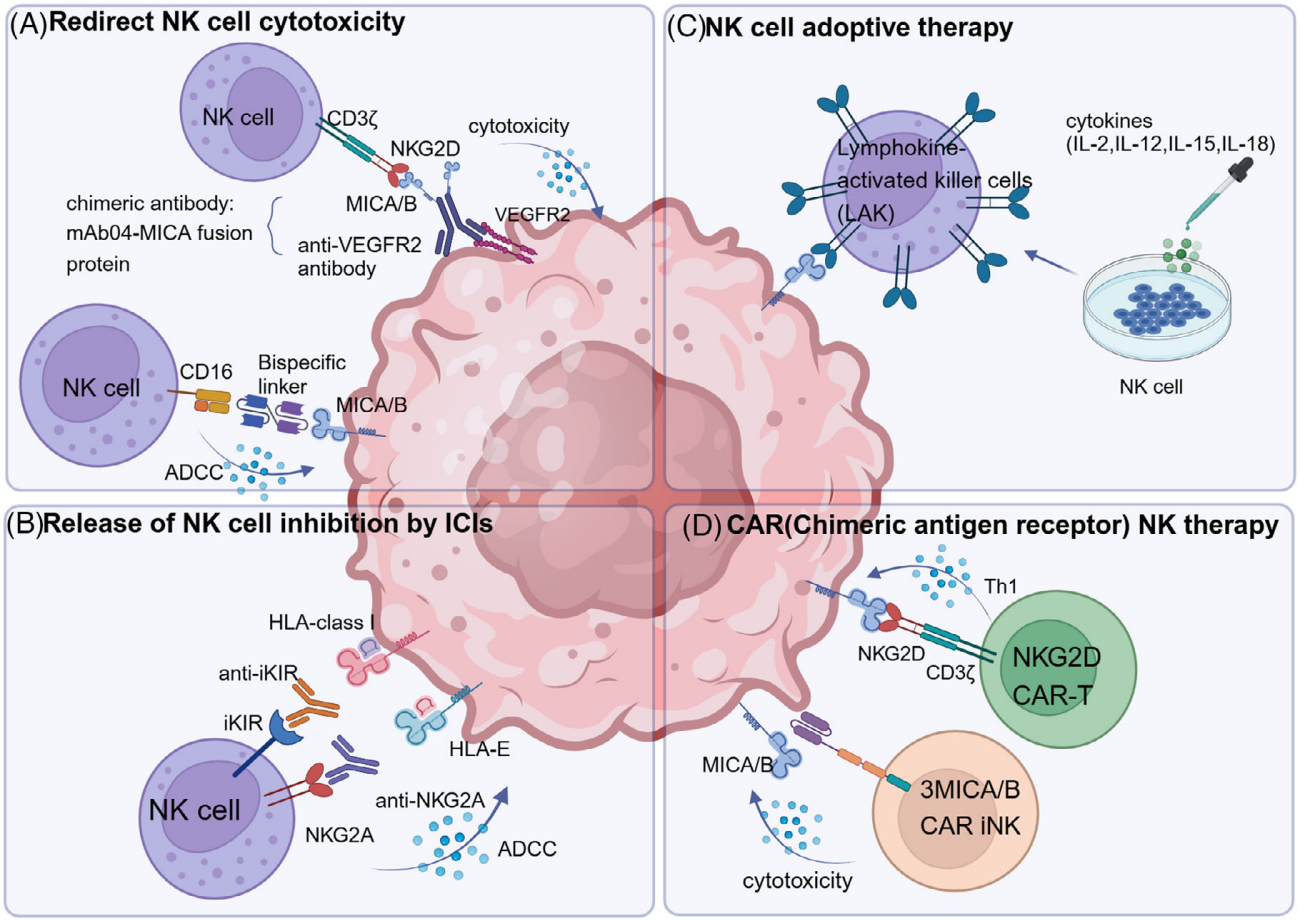
NK‐based immunotherapies. (A) Bispecific proteins (BiKE) recognize tumor antigens on one arm and bind to activating NK cell receptors on the other arm, which redirects NK cell cytotoxicity toward tumor cells and promotes the interaction between NK cells and tumor cells. Considering the power of ADCC, some bispecific proteins have been designed to provide stronger binding to CD16 than conventional antibodies. Chimeric antibody anti‐VEGFR‐MICA fusion protein cover VEGFR expression tumor cells with MICA, and sensitize them to NKG2D‐mediated killing. (B) Immune checkpoint inhibitors (ICI), which block inhibitory checkpoints with therapeutic antibodies, have greatly unleashed NK cell antitumor potential and improved cancer immunotherapy. Monoclonal antibodies that target KIR and NKG2A have been developed to block their interaction with HLA I‐class molecule on the tumor cells, improve NK cell effector functions and ADCC. (C) NK cell adoptive therapy: Infusing ex vivo cytokines (such as IL‐2, IL‐12, IL‐15, and IL‐18) can stimulate NK cells to become lymphokine‐activated killer (LAK) cells. Then, reinfuse these cells back into patients for greater cytotoxicity against malignant targets. (D) Chimeric NKG2D connects NKG2D to the CD3ζ chain on the surface of NK cells, then lyses target cells in a NKG2D ligand‐dependent manner. 3MICA/B CAR, a novel CAR targeting the conserved α3 domain of MICA/B (3MICA/B CAR), into a multiplexed‐engineered induced pluripotent stem cell (iPSC)‐derived NK cell (3MICA/B CAR iNK).

One way to redirect NK cell cytotoxicity toward tumor cells is through bispecific (BiKE) and/or trispecific engagers (TriKE) to engage an NK cell response.^238^ Bispecific proteins recognize tumor antigens on one arm and bind to activating NK cell receptors on the other arm, which promotes the interaction between NK cells and tumor cells (Figure 5A). It has been demonstrated that a CD16‐targeting and CD33‐targeting BiKE is efficacious against MDS and AML blasts in AML, especially when used in conjunction with an inhibitor of the disintegrin and MP domain‐containing protein 17 (ADAM17), which maximizes ADCC activation.^239^ In vitro, AFM24, an IgG1‐scFv fusion antibody that targets both EGFR on tumor cells and CD16 on innate immune cells, was extremely effective in triggering ADCC through NK cells.^240^ In order to improve antigen specificity and NK cell persistence in vivo, Vallera et al.^241^ have created a TriKE, which consists of two antibody fragments directed against CD16 and CD33 along with IL‐15, which can activate and expand NK cell populations. It is presently undergoing phase I trials for a variety of CD33^+^ hematological malignancies (NCT03214666).

Among immunotherapy with activated receptors, NKG2D–MICAB has been studied the most. The NK cell engagers, which contains Fab fragments binds to HER2 on tumor cells and NKG2D on NK cells, thereby inducing cytotoxicity through NK cells.^242^ In recent years, NK cell recognition of tumor cells can be enhanced by MICA α1–α2 and anti‐VEGFR2 bispecific protein.^243^, ^244^ This mAb04‐MICA fusion protein comprises full‐length human anti‐VEGFR2 antibodies and MICA α1–α2 ectodomain, which displayed antineoplastic activity through VEGFR2 and NKG2D targeting^243^, ^244^ (Figure 5A). The fusion protein enables tumor cells with VEGFR2 to be recognized by NKG2D on NK cells. It can inhibit the proliferation of tumor and tumor angiogenesis in gastric cancer and non‐small cell lung cancer, and also promote intratumoral NK and T cell infiltration and activation, resulting in efficient tumor suppression.

### 6.2 Engagement of NKG2D ligands

#### 6.2.1 Antibodies preventing the shedding of MICA/B

Recently, mAbs targeting the conserved, membrane‐proximal α3 domain of MICA/B are found to prevent MICA/B shedding and enhance NK cell antitumor efficacy. Mice are immunized with the recombinant MICA α3 domain and three mAbs (7C6, 6F11, and 1C2) are identified that bound to the α3 domain, the extracellular domain that interacts with ERp5 to initiate cleavage.^245^ Among these mAbs, 7C6 is most effective in stabilizing MICA and MICB.^164^

**FIGURE 6:**
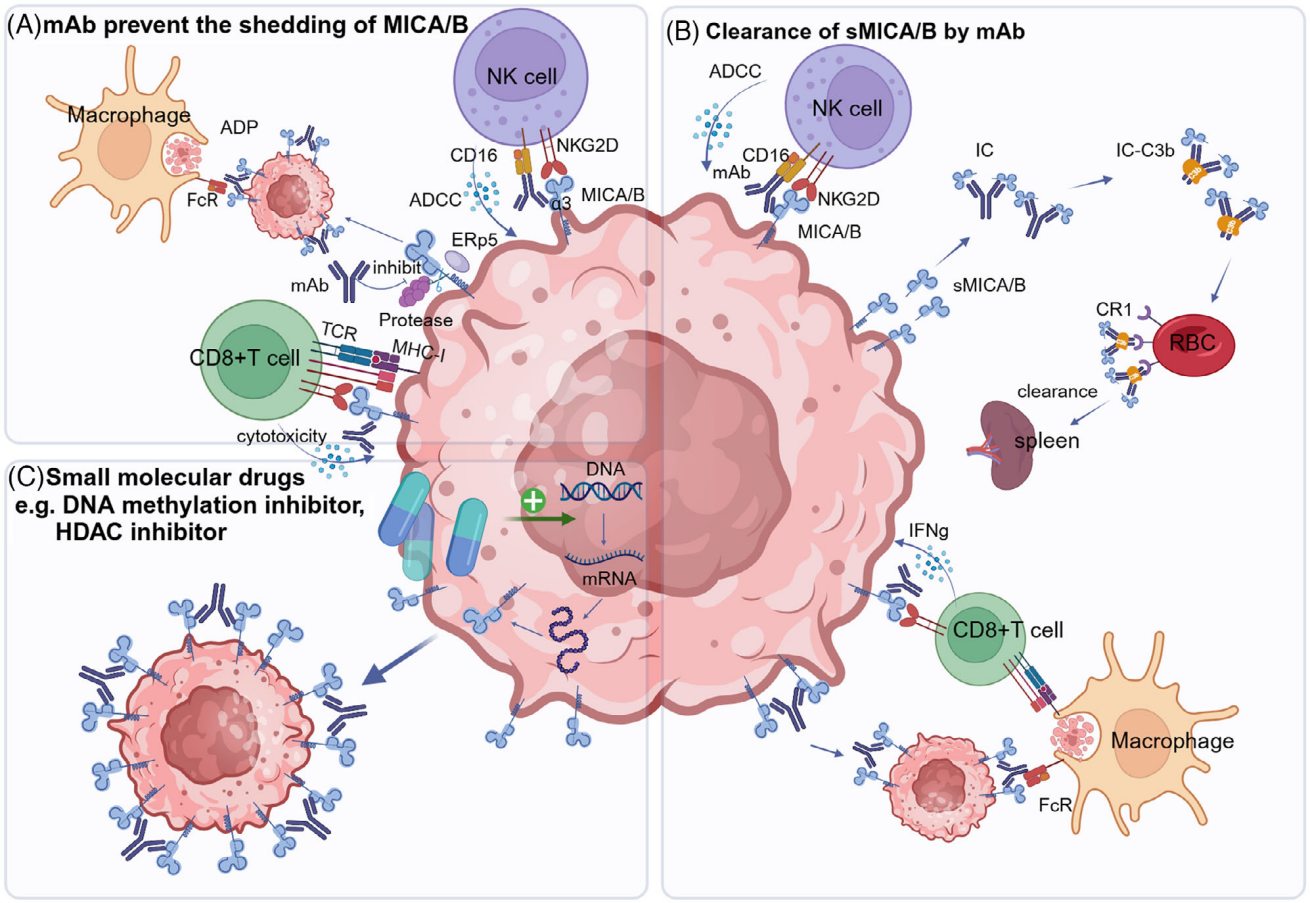
Innovative immunotherapeutic strategies targeting MICA/B. (A) Monoclonal antibodies targeting MICA/B α‐3 domain can prevent the shedding of MICA/B. The 7C6 mAb increases their cell surface density, and finally increase their binding and activation of NKG2D on NK cells and CD8+T cells. Besides, the mAb also triggers ADCC through Fc receptors on NK cells and ADP. (B) Ab‐mediated clearance of sMICA/B. B10G5, a mAb which targets at sMICA/B, could neutralize free sMICA/B, antagonize immune suppression and result in a recovery of NK and CD8+ T cell‐mediated cytotoxicity. Moreover, B10G5 trigger ADCC, and also opsonize DC to enhance antigen cross‐presenting to CD8 T cells. (C) Small molecular drugs promote MICAB transcription. Certain DNA methylation inhibitors and HDAC inhibitors could potently increase MICA/B expression on the tumor cells, thereby promoting the tumor immunity of NK cell and CD8+T cell.

Humanized 7C6 mAb (hIgG1) could activate NK cells through two important receptors, the NKG2D and CD16 Fc receptors (Figure 6A and Table 2). The mAbs could inhibit MICA/B shedding, increase their cell surface density, and finally increase their binding to NKG2D on NK cells. Meanwhile, the 7C6 mAb also triggers ADCC through Fc receptors on NK cells.^246^ Thus, 7C6 could increase NK cell cytotoxicity through stabilizing MICA/B and ADCC. Of importance, they do not obstruct NKG2D to bind to the α‐1 and 2 domains.^3^

**TABLE 2:** Therapies targeting NKG2D and MICA/B.

Category	Drug name	Target	Mechanism	References
Monoclonal antibody (mAb)	7C6	MICA/B α3 domain	Prevent MICA/B shedding and enhance NK cell antitumor efficacy	246
Antibody	B10G5	sMICA/B	Neutralize free sMICA/B	247
Epigenetic drug	Deoxycytidine	DNA	Inhibit the DNA methylation, thus increase MICA and MICB expression	248
Epigenetic drug	Panobinostat	HDAC	Inhibit HDAC and potently increase MICA/B expression	120, 162
CAR NK	3MICA/B CAR	MICA/B α3 domain	Induce NKG2D‐beared NK cells into body to improve the immune function of NK cell	249
Vaccine	MICB‐vax vaccine	MICA/B α3 domain	Induce high‐titer antibodies targeting at α3 domain, inhibit the proteolytic shedding	250
BLS‐MICA	BLS‐MICA	MICA/B full domain	Induce high‐titer antibodies targeting at α3 domain, inhibit the proteolytic shedding; interfere with a tumor‐immune escape mechanism through scavenging of sMICA from serum	251

Besides NK cells, the 7C6 mAb is proposed to activate other immune cells including macrophages and CD8+ T cells, which further promotes the antitumor immunity (Figure 6A). The 7C6 mAb binds surface MICA/B in tumor cells, which in turn are phagocytosed by macrophages on Fc receptor engagement, which is named antibody‐dependent phagocytosis (ADP).^246^ Furthermore, NKG2D is expressed by CD8+ T cells and provides costimulation.^51^, ^137^ Therefore, inhibition of MICA/B shedding may also promote CD8+ T‐cell‐driven immunity and enhance the therapeutic efficacy of T‐cell checkpoint blockers and serve as an alternative for cancer patients who are resistant to PD‐1/PD‐L1 antibodies. Combination of 7C6 and PD‐1 checkpoint therapy may exert more robust synergistic effect.

#### 6.2.2 Antibody‐mediated clearance of sMICA/B

Proteolytic proteases‐mediated tumor‐shedding of sMICA/B accounted for one of the major mechanisms for MICA/B tumor evasion of NKG2D immune surveillance.^247^ The soluble NKG2D ligands (sMICA and sMICB) have been shown to subvert antitumor immunity through multiple mechanisms, including downmodulation of NKG2D on NK and CD8+ T cells, and expansion of suppressive MDSC and M2 macrophages.^212^

B10G5 is a mouse IgG1 isotype, recognizing both MICA and MICB, thus could neutralize free sMICA/B^211^, ^252^ (Figure 6B) (Table 2). The soluble antigen–antibody complex (IC) binds to C3b, and adheres to the surface of RBCs and platelets then phagocytosed and cleared by macrophages in the spleen, thus rescue the tumor immune suppression induced by sMICA/B.^253^ Recently, researchers have generated a “humanized” mouse model that expresses human MICA/B. The model has successfully demonstrated that therapy with the sMICA/B nonblocking monoclonal B10G5 can effectively induce regression of advanced primary tumors and eliminated metastasis.^247^ The therapeutic effect is conferred by unleashing endogenous antitumor immune responses.

B10G5 and NKG2D recognizes different epitopes of MICA/B,^247^ thus B10G5 does not block the NKG2D‐mediated NK cell cytotoxicity. It promotes the NKG2D‐MICA/B connection by ADCC, thus further enhance susceptibility of MICA/B‐tumor cells to NK cell killing.^247^ B10G5 also significantly increased the population of NKG2D+ CD8 T cells in the periphery, and revived cytotoxic CD8 T‐cell antitumor responses (Figure 6B). In addition, it could potentiate CD4 T cells to Th1 responses in the tumor dLNs, resulting in significant increase in CD4 T cells with CD44hi memory phenotype. These effects may function through opsonized phagocytosis by dendritic cells and enhanced antigen presentation and cross‐presenting to CD4 and CD8 T cells.^254^, ^255^ Furthermore, B10G5 also could eliminate arginase I+ immune suppressive myeloid cells in tumor parenchyma.^212^

Recently, it has been shown that Ab‐mediated blockade of CTLA4 in mouse models could boost antitumor immunity in patients with melanoma.^256^, ^257^ Notably, administration of anti‐CTLA4 mAb spontaneously induced anti‐MICA antibodies, which could clear soluble MICs, antagonize immune suppression and enhance innate and adaptive antitumor cytotoxicity. Thus, antibody‐mediated clearance of soluble MICA/B is also involved in other immunotherapeutic strategies.

#### 6.2.3 Small molecules promoting MICA/B transcription

As MICA/B expressing tumor cells are more sensitive to NK cytotoxicity, many epigenetic drugs are found to increase MICA and MICB expression and exert antitumor effects (Figure 6C). Deoxycytidine, a DNA methylation inhibitor, could increase the cell‐surface MICB expression and sensitize the cells to NK cell‐mediated cytotoxicity^248^ (Table 2). HDAC inhibitors also potently increase MICA/B expression by several cancer types, thereby promoting NK‐cell‐driven immunity.^258^, ^259^, ^260^, ^261^ Panobinostat and romidepsin, two broad‐spectrum HDAC inhibitor, increased MICB mRNA in AML cells and enabled subsequent stabilization of the translated MICB protein by 7C6. Combined use of 7C6 and panobinostat substantially increased surface MICA/B expression in human AML cells^120^, ^162^ (Table 2).

Several other drugs could also increase MICA/B expression by human cancers. Dacarbazine could upregulate the NKG2D ligands on tumor cells to activate NK and CD8 T Cells and restrain melanoma growth.^262^ Poly (ADP‐ribose) polymerase 1 inhibitors could induce the expression of NKG2DLs on leukemic stem cells to inhibit AML recurrence.^263^ In addition, proteasome inhibitors could augment the NKG2D ligand expression in multiple myeloma.^264^ All of them can be combined with 7C6 anti‐MICA/B antibodies to eliminate tumors by activating NK cell and converting “cold” tumors to “hot” tumors.^109^

### 6.3 Release of NK cell inhibition

NK cell function is often restricted by signaling through inhibitory receptors and immunological checkpoints; hence, inhibiting these pathways can unleash NK cell antitumor potential. Clinical‐grade mAbs that target KIR and NKG2A have been developed with the intention of enhancing or unleashing the antitumor NK cell function (Figure 5B).

Lirilumab, a mAbs targeting KIRs (specifically inhibitory KIR2DL1, KIR2DL2, and KIR2DL3), is a fully human IgG4 antibody and has demonstrated therapeutic potential in preclinical rodent models of AML^265^ and multiple myeloma in synergistic with lenalidomide.^266^ Despite favorable results from phase I clinical trials, Lirilumab as monotherapy did not increase leukemia‐free survival in elderly patients with AML, according to recently published data from the French study EFFIKIR.^267^ Currently, more preclinical and clinical research is required to identify the best ways of Lirilumab therapy, as well as its indications.

As an increasingly recognized immunological checkpoint, NKG2A and its ligand HLA‐E have been the focus of mAbs used in cancer immunotherapy. Monalizumab, an IgG4 blocking mAb against NKG2A, improves NK cell effector functions and stimulates effector T cell responses combined with anti‐PD1.^268^ S095029, a new Fc‐silenced NKG2A‐blocking antibody with clinical development potential,^269^ counteracts the inhibitory effects of the NKG2A/HLA‐E interaction in multiple experimental models. Phase 1 dose escalation studies are presently being conducted to assess S095029 as a single drug or in conjunction with anti‐PD‐1 therapy (NCT05162755).^269^ According to Ghaffari's research, a TCR mimic antibody called EXX‐1 that binds to the NKG2A ligands had encouraging antitumor properties.^270^ In addition to inhibiting the NKG2A pathway, EXX‐1 Fc antibody probably causes tumor cell death via ADCC.

### 6.4 NK cell adoptive therapy

Due to the potent killing activity of NK cells, NK cell adoptive transfer therapy has emerged as a prominent focus in the field of tumor immunotherapy. It involves the infusion of ex vivo activated and expanded NK cells to enhance antitumor immune responses. NK cells can be obtained from the patient (autologous setting) or from a healthy donor (allogeneic setting).^271^

Activating endogenous NK cells and encouraging their proliferation in patients were the goals of early research aiming to increase the anticancer activity of NK cells. Autologous NK cell adoptive transfer has been explored in various malignancies, including lymphoma, leukemia, and solid tumors.^272^ It has the advantage of reducing the risk of graft‐versus‐host disease (GVHD) and immune rejection.

One major strategy was to infuse ex vivo cytokines (such as IL‐2, IL‐12, IL‐15, and IL‐18), which stimulates NK cells to become lymphokine‐activated killer (LAK) cells and then to reinfuse these cells to exhibit greater cytotoxicity against malignant targets^273^, ^274^, ^275^ (Figure 5C). It has been demonstrated that combining IL‐2 and IFN‐α with GM‐CSF is beneficial to further increase the activity of NK cells, offering a strong foundation for the use of IL‐2 to activate endogenous NK cells' anticancer activity.^273^ However, endogenous NK and LAK cells may not have enough cytotoxicity to fight tumor cells that have progressed.^276^ To effectively guide autologous NK cells to kill tumor cells, it is therefore necessary to develop a mechanism to circumvent the inhibition of autologous NK‐cells by self‐HLA molecules.

Allogeneic NK cell therapy has the advantage of providing a large and potentially more potent NK cell population. This strategy enables the use of NK cells from healthy donors who may have enhanced cytotoxicity and can bypass inhibitory signals from tumor cells. Since tumor cells lack the proper MHC class I ligands to bind inhibitory KIRs, they are more prompt to be destroyed by allogeneic NK cells.^277^ This approach has shown promise in hematological malignancies and solid tumors.^278^ One phase I clinical trial found that adoptive transfer of allogeneic NK cells grown and activated in vitro with IL‐15 and hydrocortisone (HC) was safe and potentially effective when used in patients with advanced non‐small cell lung cancer in conjunction with standard treatment.^279^

The development of NK cell adoptive transfer therapy has shown promising results in the field of cancer immunotherapy. Clinical trials have demonstrated its safety, feasibility, and potential efficacy. However, challenges such as optimizing NK cell expansion, insufficient cytotoxicity, and immune‐mediated rejection due to MHC mismatch remain to be addressed. Future research will focus on refining the therapeutic strategies and combinations with other immunotherapies to maximize the potential of NK cell adoptive transfer therapy in treating cancer.

### 6.5 CAR‐NK cells and NKG2D CAR‐T therapy

CAR‐T/NK is a rapidly developing adoptive immunotherapy of tumor in recent years. This therapy introduces synthetic CARs into T/NK cells to enable them to specifically identify and attack tumor cells. The outcomes of recent clinical study indicate that CAR‐NK therapy has higher benefits.^280^, ^281^ Infusions of allogeneic CAR‐NK cells can lower the risk of GVHD that results from the response of allogeneic T cells against the host tissues of recipients who are immunosuppressed.^282^ Furthermore, NK cells themselves are difficult to induce excessive cytokine secretion, which makes neurotoxicity and cytokine release syndrome less common in CAR‐NK immunotherapy.^283^, ^284^ In addition, CAR‐NK therapy does not require the patient's autologous immune cells as the source, and the advantages of lower treatment cost both may make it a major competitor of CAR‐T therapy.^280^

Research on CAR‐NK cells has mostly used CAR designs intended for CAR‐T cells thus far. New CAR constructions have recently been created especially for NK cells.^285^, ^286^ However, the majority of related trials focus on hematological malignancies using CAR‐NK cells to target CD19, CD22, and B cell maturation antigen (BCMA).^287^ Recently, the first large‐scale CAR‐NK cell trial (NCT03056339) demonstrated that anti‐CD19 CAR NK‐cell therapy has shown remarkable clinical efficacy in B‐cell cancers and had the potential to overcome these limitations of CAR T‐cell therapy.^283^ Imai et al.^287^ reported that a second‐generation anti‐CD19 CAR containing 4‐1BB costimulatory domain (scFv‐CD8TM‐4‐1BB‐CD3ζ), which overcame inhibitory signals and induced NK cell specific killing of CD19+ acute lymphoblastic leukemia. Similarly, a second‐generation CAR targeting BCMA has demonstrated significant anti‐MM activity in vitro and in vivo.^288^ To address the issue of antigen escape and achieve a more durable response, a dual‐targeted CAR‐NK cell therapy has been proposed, targeting both BCMA and GPRC5D.^289^, ^290^ A high expression of human EGFR 2 (HER2) in breast, renal cell and GBM cancer makes it an ideal candidate to develop immunotherapy using HER2‐CAR‐modified NK cells.^291^, ^292^, ^293^ Then CAR construct containing CD28 costimulatory domain has also been developed to direct against HER2.^294^

NKG2D ligands are primarily expressed on tumor cells but are absent on most normal tissues. Chimeric NKG2D connects NKG2D to the CD3ζ chain on the surface of T cells (Figure 5D). These chimeric NKG2D (chNKG2D)‐modified T cells produced large amounts of T‐helper 1 cytokines and lysed target cells in a NKG2D ligand‐dependent manner.^295^ Soluble MICA/B might desensitize the engineered T cells potentially by downregulating the chimeric NKG2D receptor. However, chNKG2D T cells are resistant to inhibition by high concentrations of sMICA. When exposed to 1.5 ug/mL of soluble MICA, engineered T cells are still not deactivated, which further enhances the feasibility of this strategy.

Furthermore, in the treatment of ovarian cancer, valproate increases NKG2DL on the surface of cancer cells, and enhances the lethality of CAR‐T cell.^296^ Thus, we can further enhance NKG2D‐CAR‐T cell's cytotoxicity against tumors through adjuvant methods, which could increase the expression of NKG2DL on tumor cells (Table 1). Of note, chimeric NKG2D receptor T cells were well tolerated by patients with hematological malignancies in a clinical trial reported recently, thus serving as the first evidence of the safety of an NKG2D‐based CAR‐T immunotherapy.^297^ Meanwhile, such chNKG2D CAR‐NK cells may also be functional in efficient tumor killing^298^ (Table 1).

Besides NKG2D itself, chimeric antibodies targeting NKG2D ligands are also employed to construct CAR‐T cells. According to a recent study,^249^ researchers incorporated 3MICA/B CAR, a novel CAR targeting the conserved α3 domain of MICA/B (3MICA/B CAR), into a multiplexed‐engineered induced pluripotent stem cell (iPSC)‐derived NK cell (3MICA/B CAR iNK) (Figure 5D and Table 2). 3MICA/B CAR mitigates MICA/B shedding and inhibition via soluble MICA/B, while simultaneously shows potent cytolytic activity against various solid and hematological tumor models. The data demonstrate a promising pan‐cancer immunotherapy approach.

However, due to CAR‐NK cells' short lifespan in the bloodstream, there is comparatively little chance of on‐target or off‐tumor damage to normal organs.^299^ And for solid tumors, they may also have some restrictions.^300^ It is primarily because of the immunosuppressive TME, and weak capacity of NK cells to infiltrate solid tumors.^301^ Thus, antitumor adoptive cell immunotherapy of NK cells and NKG2D CAR‐T/NK cells may need to be combined with other strategies in order to provide an efficient antitumor function.^109^

### 6.6 Innovative tumor vaccines targeting MICA/B

Therapeutic cancer vaccines have undergone a resurgence in the past decade. The purpose is to stimulate the patient's adaptive immune system against specific tumor antigens to regain control over tumor growth, induce regression of established tumors and eradicate minimal residual tumors.^302^ Current therapeutic cancer vaccines are mainly based on neoantigens produced by specific mutations in tumor cells, necessitating personalization owing to the vast diversity in MHC molecules that present peptides to T cells.^60^ In addition, the inactivating mutations (or downregulation) of genes in the MHC‐I antigen presentation or IFN‐γ signaling pathways sometimes greatly impair CD8+ T cell‐mediated tumor immunity, and gives rise to the resistance of tumor vaccine.^234^, ^235^, ^236^ Recently, the innovative antitumor vaccines targeting MICA/B have been developed, and may possess a good prospect on tumors, especially those have defects in MHC class I antigen presentation pathway.

**FIGURE 7:**
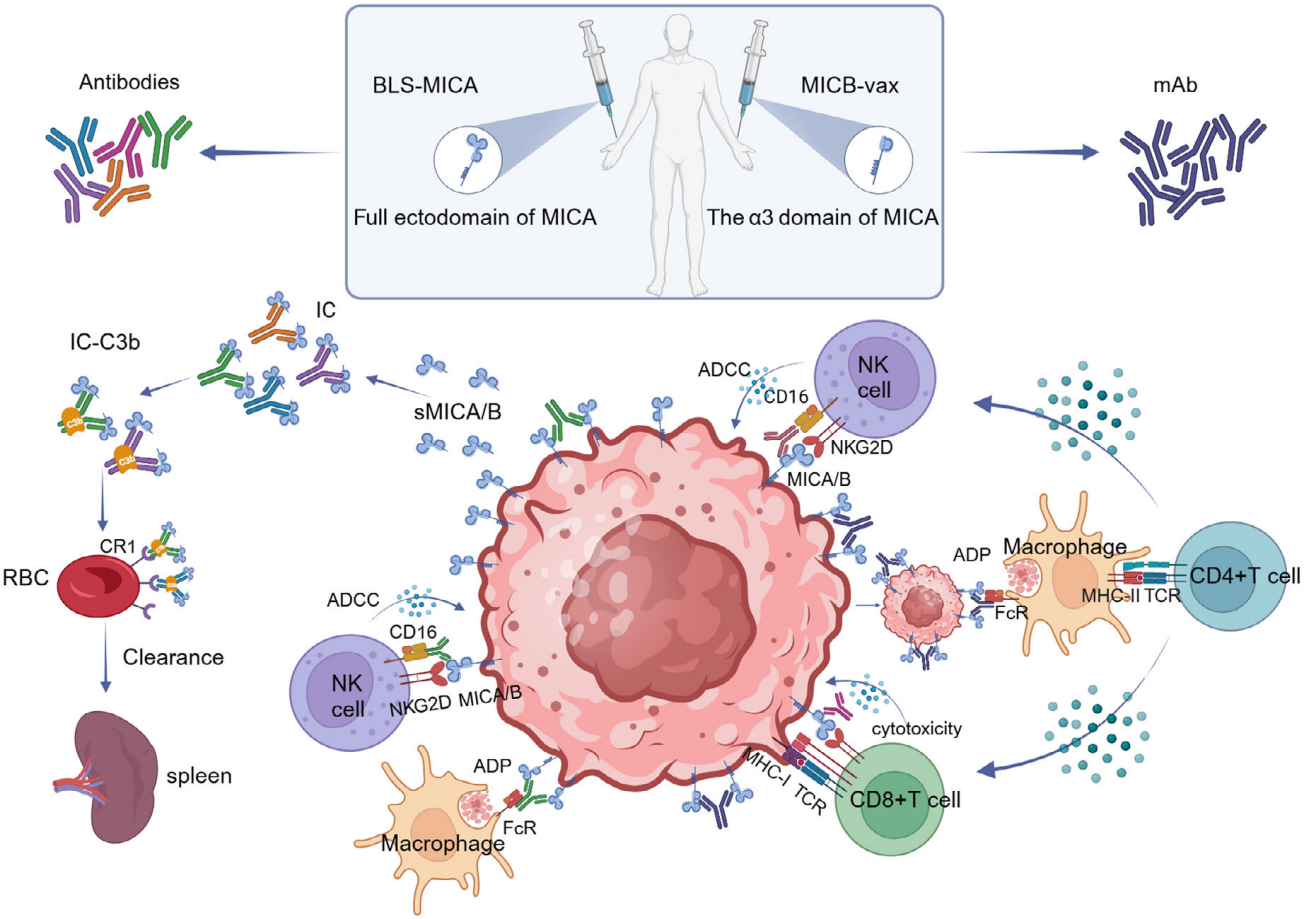
Innovative antitumor vaccines targeting MICA/B. The specific vaccine MICB‐vax induce specific antibodies targeting the highly conserved α3 domain in MICB. BLS‐MICA is a chimeric protein consisting of the full ectodomain of MICA fused to highly bacterial immunogenicity protein BLS, which could induce high‐titer anti‐MICA antibodies in vivo. The antibodies targeting α3 domain inhibit proteolytic shedding of MICA/B, and increased MICA/B on the surface of tumor cells, which could bind and active the NKG2D receptors on NK cells and CD8+T cells. Antibodies targeting α1 and α2 can interfere with a tumor‐immune escape mechanism through scavenging of sMICA from serum. All these antibodies bind on tumor cells further promote tumor cytotoxicity through ADCC and ADP and also enhance presentation of tumor antigens by antigen‐presenting cells (dendritic cells, etc.) to CD4+ and CD8+ T cells. These vaccines could finally result in a recovery of NK and CD8+ T cell‐mediated cytotoxicity and a better prognosis of the disease.

A new universal therapeutic cancer vaccine has been invented recently, which targets NKG2D–NKG2DL pathway instead of neoantigens, and it is suitable for all kinds of tumors.^60^ It also maintains efficacy against MHC I‐deficient tumors, which are resistant to cytotoxic T cells, through the coordinated action of NK cells and T cells.^60^ This specific MICB‐vax vaccine applies the α3 domain of MICB to induces high‐titer antibodies targeting the highly conserved α3 domain (Figure 7), then the antibodies strongly bind to tumor cells expressing human MICB and prevent its shedding.^250^ Subsequently, dendritic cells that recognize antibody‐bound tumor cells through FcR are activated, internalizing and presenting antigens to T cells, and further promote the recruitment of NK cells.^250^ The recruited NK cells and CD8+T cells become activated by binding to MICA/B on the surface of tumor cells, exerting the tumor killing effect of the immune system.

MICB‐vax not only controlled the established tumors, but also greatly reduced the number of lung metastases in melanoma and breast cancer models^60^ (Table 2). This vaccine also shows substantial efficacy in controlling subcutaneous EL4 tumors that expressed either MICB or MICA.^60^ MICB‐vax also could induce immunological memory to prevent tumor recurrence. Furthermore, this vaccination also successfully controlled tumors lacking β2m, which have impaired TCR recognition and killing via CD8+ T cells. Both CD4+T cells and NK cells are also necessary in the efficacy.^60^ Now a first‐in‐human clinical trial with MICB‐vax is being planned.

Brucella spp lumazine synthase (BLS)‐MICA constitutes another novel antitumor vaccine^251^ (Table 2). It is a chimeric protein consisting of the full ectodomain of MICA fused to highly bacterial immunogenicity protein BLS that display intrinsic adjuvant properties and induce high‐titer anti‐MICA antibodies in vivo.^251^ The use of BLS‐MICA as vaccine to induce therapeutic anti‐MICA pAb constitutes a “all‐in‐one” strategy (Figure 7). The specific antibodies that target at MICA α3 domain could inhibit the proteolytic shedding of MICA/B and then activate NKG2D ligands on NK cells and CD8+T cells. In addition, BLS‐MICA could induce antibodies recognize α1 and α2, which simultaneously scavenging sMICA from serum. Furthermore, all these antibodies also promote tumor elimination through ADCC and ADP. By these mechanisms, it could lead to a reprogramming of the tumor microenvironment toward an proinflammatory phenotype and finally result in a recovery of NK and CD8+ T cell‐mediated cytotoxicity and a better prognosis.^254^, ^303^ However, it should be taken into consideration that blocking antibodies that generated by BLS‐MICA may impair the recognition of MICA/B expressing tumors by NK cell and reducing the tumor surveillance.

### 6.7 Immune modulation drugs targeting NK cells

Despite the fact that NK cells identify and eradicate tumor cells in vitro, the tumor microenvironment is crucial in determining their antitumor efficiency in vivo. Thus, cytokines and drugs targeting TME and NK cells also showed potential effects in NK cell‐mediated tumor immunotherapy.

IL‐2 has been identified as key cytokines that upregulate the activity of NK cells. However, it has the side effects that it drives Treg cell development, which produces immunosuppressive cytokines on tumor‐infiltrating lymphocytes. More recently, IL‐2 variants have been designed that induce the expansion of effector immune cell populations but promote only minor expansion of the Treg cell population.^304^, ^305^ An alternative method entails introducing engineered synthetic IL‐2 (OrthoIL‐2) into constructed T cells. This IL‐2 is only able to communicate with its matching engineered receptor (OrthoIL‐2R).^306^

When compared with IL‐2, IL‐15 might be a preferable choice because it has the ability to boost NK cell populations and their activating receptor expression without stimulating the growth of Treg cell populations. A phase I clinical trial of patients with metastatic malignancies has reported that daily infusion of IL‐15 induces NK cell proliferation and substantially increases the number of NK cells.^307^

In addition to stimulatory cytokines, inhibitory factors in the tumor microenvironment can hinder NK cell function, with TGF‐β being a major suppressor of NK cell responses.^308^, ^309^ Phase I studies are now testing vactosertib (TEW‐7197), an oral bioavailable inhibitor of the serine/threonine kinase TGFβ receptor type 1 (TGFR‐1), as a monotherapy for advanced solid tumors (NCT02160106) and MDS (NCT03074006). Galunisertib is another TGFR‐1 kinase inhibitor that has shown promise in treating AML and colon cancer.^310^ Its administration is linked to the production of TNF and IFN‐γ, as well as the restoration of NKG2D expression on NK cells.

Moreover, novel medication classes that possess both immunomodulatory and direct antitumor actions can regulate the function of NK cells.^311^ Thalidomide analogs (such as lenalidomide and pomalidomide) called “immunomodulatory drugs” are of special interest because of their ability to increase NK cell‐mediated cytotoxicity by increasing NCR expression, expanding NK cell populations and increasing immune cell recognition of tumor cells in various models.^312^ For instance, one study found that lenalidomide increased NK cell cytotoxicity and IFN‐γ production while simultaneously decreasing the immunosuppressive activities of Treg cells.^313^

Recently, a new study proposed that the use of inhibitors targeting sphingomyelin can significantly increase the sphingomyelin content of NK cell membrane in tumor microenvironment and restore NK cell membrane protrusions, thus improving tumor cell recognition and killing ability. Intervention targeting sphingomyelin enzyme combined with immune checkpoint blockers has a synergistic anticancer effect.^314^

## 7 CONCLUSIONS AND PERSPECTIVE

NK cells have enormous therapeutic potential and are currently a key component of the tumor immunotherapy area. The damage caused by a tumor can be significantly decreased as long as the pertinent molecules involved in tumor immune escape are found and altered.

Nowadays, choosing novel target molecules and therapeutic approaches is a crucial path. With NKG2D‐dependent NK cell‐mediated anticancer effects, targeting the NKG2D/NKG2DL axis, and particularly MICA and MICB within it, is a very appealing target for tumor immunity promotion. Adoptive cell treatments, vaccines, and antibodies related to this axis have previously been produced. Antibodies against MICA/B are currently known to have noticeable effects on hematological malignancies,^246^ but treating solid tumors still presents a number of challenges, particularly when trying to overcome the immunosuppressive TME. Furthermore, even though MICA/B is mostly expressed on cancerous cells, toxicity must still be taken into account because these ligands may be produced by a variety of stressors.

Nevertheless, treatments that target NKG2D/NKG2DL represent a cutting‐edge, novel criterion in tumor immunotherapy, offering a multitude of opportunities and potentially having a significant antitumor effect when combined with other treatments. Tumor clearance may be enhanced and synergistic effects may result from combining NK cell‐based therapies with other immunotherapies such immune checkpoint inhibitors or CAR‐T cell therapy. Additionally, it can be used in conjunction with a number of traditional treatments, including targeted therapies, radiation therapy, and chemotherapy, to offer a more all‐encompassing approach to the treatment of cancer. NK cells can help eliminate residual tumor cells and prevent immune evasion after these treatments. What is more, we can enhance NK cell effector function functionality through genetic modification of NK cells,^315^, ^316^ overexpress specific activating receptors or cytokines, or by improving NK cell expansion and persistence in vivo. Additionally, we can modulate immunosuppressive signals in the tumor microenvironment, such as strategies targeting inhibitory receptors, such as PD‐1, NKG2A, or TGF‐β, could enhance NK cell functionality and counteract immune evasion by tumor cells.

Overall, further research and development are needed to optimize NK cell and NKG2D‐based therapies for effective tumor immunotherapy. Combination approaches, targeting immunosuppressive signals, and genetic engineering techniques hold promise for enhancing NK cell antitumor responses and improving patient outcomes.

## AUTHOR CONTRIBUTIONS

DanRu Wang, LiHao Sui, and LingYun Dou drafted the paper and prepared figures. Yiquan Xue and Sheng Xu reviewed and edited the manuscript. All authors have read and approved the final manuscript.

## CONFLICT OF INTEREST STATEMENT

The authors declare no conflict of interest.

## ETHICS STATEMENT

Not applicable.

## Data Availability

Not applicable.
